# Multi-channel acoustic recording and automated analysis of *Drosophila *courtship songs

**DOI:** 10.1186/1741-7007-11-11

**Published:** 2013-01-31

**Authors:** Benjamin J Arthur, Tomoko Sunayama-Morita, Philip Coen, Mala Murthy, David L Stern

**Affiliations:** 1 Department of Neurobiology & Behavior, Cornell University, Ithaca, NY 14850, USA; 2 Janelia Farm Research Campus, Howard Hughes Medical Institute, Ashburn, VA 20147, USA; 3 Howard Hughes Medical Institute, Princeton University, Princeton, NJ 08544 USA; 4 Department of Ecology & Evolutionary Biology, Princeton University, Princeton, NJ 08544 USA; 5 Princeton Neuroscience Institute, Princeton University, Princeton, NJ 08544, USA; 6 Department of Molecular Biology, Princeton University, Princeton, NJ 08544 USA

**Keywords:** courtship song, *Drosophila*, evolution, genetics, multi-channel recording, neurobiology, song segmentation software

## Abstract

**Background:**

*Drosophila melanogaster *has served as a powerful model system for genetic studies of courtship songs. To accelerate research on the genetic and neural mechanisms underlying courtship song, we have developed a sensitive recording system to simultaneously capture the acoustic signals from 32 separate pairs of courting flies as well as software for automated segmentation of songs.

**Results:**

Our novel hardware design enables recording of low amplitude sounds in most laboratory environments. We demonstrate the power of this system by collecting, segmenting and analyzing over 18 hours of courtship song from 75 males from five wild-type strains of *Drosophila melanogaster*. Our analysis reveals previously undetected modulation of courtship song features and extensive natural genetic variation for most components of courtship song. Despite having a large dataset with sufficient power to detect subtle modulations of song, we were unable to identify previously reported periodic rhythms in the inter-pulse interval of song. We provide detailed instructions for assembling the hardware and for using our open-source segmentation software.

**Conclusions:**

Analysis of a large dataset of acoustic signals from *Drosophila melanogaster *provides novel insight into the structure and dynamics of species-specific courtship songs. Our new system for recording and analyzing fly acoustic signals should therefore greatly accelerate future studies of the genetics, neurobiology and evolution of courtship song.

## Background

Many animals rely on acoustic signals to communicate both social and sexual information. Single individuals can produce highly dynamic signals, even during a single bout of communication, and social signals, particularly courtship songs, evolve quickly [1]. What genetic and neural mechanisms produce such seemingly complex patterns? Answering this question requires not only tools for genetic and neural circuit manipulation, but also sensitive assays to measure and quantify animal sounds from many individuals rapidly.

*Drosophila *courtship song has been studied extensively since Shorey discovered it in 1962 [2]. Among species that sing, song varies both qualitatively and quantitatively between species [3-5]. *D. melanogaster *courting males usually stand to the side or behind females and produce song by extending and vibrating one wing at a time. Singing males alternate between producing trains of pulses and trains of approximately sinusoidal ('sine') song; these pulse and sine trains are typically concatenated into bouts. The *Drosophila *wing is small relative to the wavelength of sound it produces by vibration, and thus the wing produces pressure waves inefficiently. On the scale of millimeters, which is approximately the distance between the singing male and the listening female, the particle velocity component of the sound, however, is larger, and the *Drosophila *female detects this sound component via her feathery arista, which is attached to the third segment of the antenna [6]. Movement of the third antennal segment activates mechanosensory neurons housed within the antenna that transmit auditory information to the brain [7,8].

Previous investigators have characterized *Drosophila *courtship song either through visual or audio recordings of a limited number of animals and, typically, investigators performed manual annotation of fly songs (for example [3,7,9-11] but see [12-14]). Human annotation has several obvious problems, including bias, fatigue-induced errors and the production of small datasets, providing limited power for statistical inference. In addition, scoring of wing extension in video recordings provides insufficient resolution to capture even the gross structure of song, such as the difference between pulse and sine trains, let alone more subtle characteristics. Many previous audio recordings have suffered from a low signal-to-noise ratio (SNR)--due to a combination of suboptimal electronics, inappropriate microphones, and/or insufficient soundproofing of the recording chamber--such that in some studies the lower amplitude components of song could not be detected reliably [13].

Here, we describe a 32-channel recording system that provides sufficient SNR to detect most song. This system can be assembled from standard electronics components and from simple recording chambers and associated parts, and we provide detailed instructions for construction and testing of the apparatus (Additional file 1). We also describe and provide open-source software that allows automated segmentation of courtship song. These resources enable recording and analysis of several orders of magnitude more courtship song data than has been available previously. We demonstrate the power of this approach by analyzing over 18 hours of courtship song, including 18,975 song bouts, from five strains of *D. melanogaster*, which allowed us to discover several previously undetected patterns. In addition, although many previous studies have characterized *Drosophila *song as relatively simple, we found that the songs of *D. melanogaster *exhibit extraordinary complexity and extensive genetic variation among natural strains for almost every aspect of song.

## Results

### Hardware

We sought to develop a multi-channel recording system with sufficient sensitivity to detect *Drosophila *courtship songs. Since 1962 [2], multiple hardware combinations have been developed for recording *Drosophila *courtship song (Table S2 in Additional file 2). We reviewed microphone type, amplifier design and chamber size used in these previous studies to identify elements of successful components that could be employed in a multiplexed system, and we chose to work with pressure-gradient microphones (Figure S1 in Additional file 3). Bennet-Clark [6,15] emphasized the advantages of detecting *Drosophila *song using pressure-gradient microphones (which indirectly detect particle velocity) versus pressure-sensitive microphones. Because particle velocity is directional, oblique sounds are attenuated, and because particle velocity falls off as 1/r^3^, the intensity of nearby sound is considerably greater than distant sound. Thus, by using pressure-gradient microphones, extraneous laboratory sounds can be reduced significantly with simple shielding. We have found that the amplitude of the noise detected by the microphones when the apparatus is placed within an acrylic box (design provided in Additional file 1) and then on an air table is indistinguishable from the amplitude of the noise measured within a soundproof chamber.

**Figure 1:**
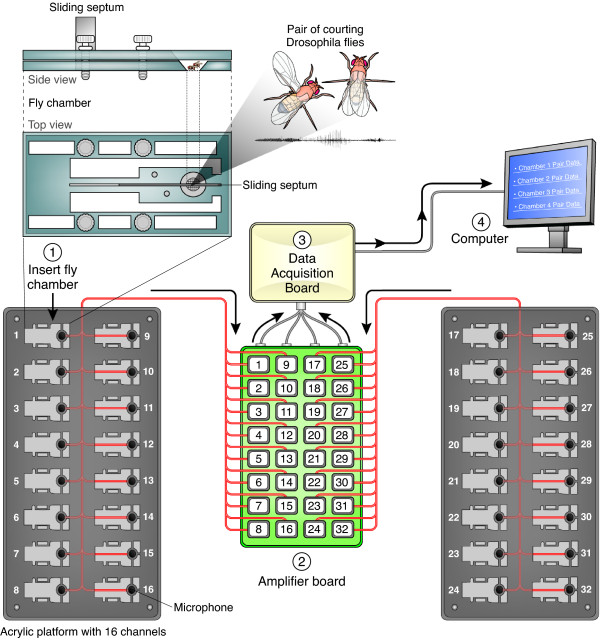
**Diagram of 32-channel courtship song recording apparatus**. A male and female are introduced into the courtship arena, which has a 5 mm diameter floor, through 2 mm holes in the top piece on either side of a movable steel septum. The chamber is fitted into the acrylic platform, positioning the flies immediately above the 9.7 mm diameter microphone, and the septum is slid back to allow the male and female to interact. Signals from the 32 microphones are amplified on a custom circuit board, converted to digital signals with a National Instruments Data Acquisition Board and recorded on the computer.

We designed a custom electronic circuit to power the microphones and to amplify and filter their output signal (Figure S2 in Additional file 3), and we designed courtship chambers to position singing males as close to the microphones as possible (Figure 1 and Figures S3 and S4 in Additional file 3). We adopted a sloped-floor design [16] to discourage flies from crawling on the ceiling of the chamber. Our chamber allows pre-loading of a single male and single female into either side of a chamber separated by a sliding septum. Flies can be recovered live after recording by placing the chamber on a diffusive CO_2 _pad to anaesthetize flies.

### Software

**Figure 2:**
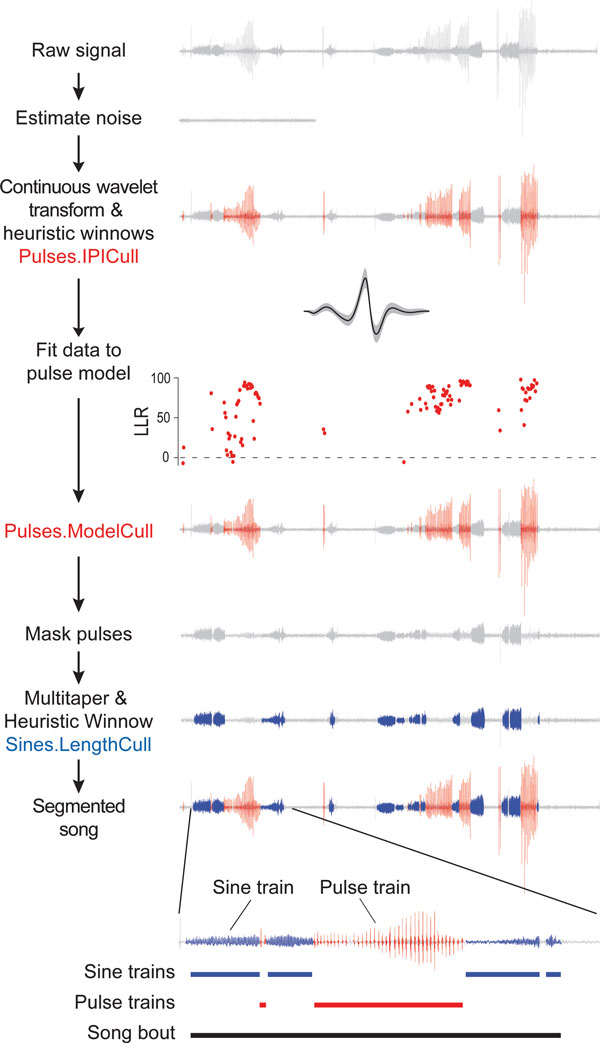
**Outline of the computational analysis performed by *FlySongSegmenter***. From top to bottom, the noise floor is estimated from the raw signal. Wavelet analysis is performed to identify putative pulses. We apply two levels of heuristic winnowing, a very conservative winnow based only on amplitude (Pulses.AmpCull) and a stricter winnow that includes amplitude winnowing and that has been tuned to *D. melanogaster *song (Pulses.IPICull); both results are provided as output. To further refine putative pulses, the log likelihood ratio is then calculated for a *D. melanogaster *model of pulses versus a model of white noise. One principled way to winnow is to retain only pulses with a log likelihood ratio > 0 (above dotted line), as shown (Pulses.ModelCull). To identify sine trains, the putative pulses are masked and multitaper spectral analysis is applied to masked data (Sines.LengthCull). All of the above steps are performed with a single call to the software and the software can be parallelized easily on a computer cluster. Modeling software is provided with the package to allow users to generate new pulse models from their own recordings. Pulse trains must contain at least two consecutive pulses and song bouts are continuous periods of alternating sine and pulse trains, separated by at least 0.5 seconds from another song bout.

**Figure 3:**
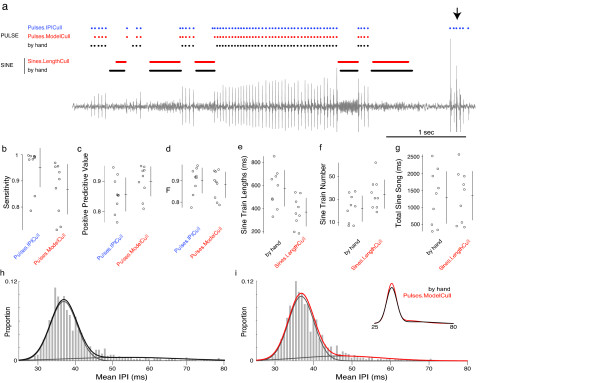
**Comparison of *FlySongSegmenter *with hand-annotated song**. We examined the accuracy of *FlySongSegmenter *by comparing automated segmentation with manual segmentation (manual annotation was performed on 60 seconds of data starting at minute 5 of a recording from each of ten males). **(a) **Approximately 5 s from a recording from one male showing pulses detected by *FlySongSegmenter *(blue and red) and manually (black) and sine trains detected by *FlySongSegmenter *(red) and manually (black). False positive pulses (arrow) are typically removed by winnowing with the pulse model. **(b-d) **The pulses found before and after model-based culling by *FlySongSegmenter *were compared to those identified manually (see Methods for more information on these measures). Culling pulses with the pulse model reduced sensitivity (b), but increased the positive predictive value (c). Overall, all methods of culling pulses resulted in similar, high overall accuracy, as measured by the F-score (d). **(e-g) ***FlySongSegmenter *often estimated shorter (e) and more (f) sine trains than manual annotation. Overall, FlySongSegmenter estimated approximately equal amounts of total sine song as manual annotation did (g). **(h, i) **Histograms of inter-pulse interval distributions (pooled across n = 10) of all pulses detected manually (h) or reported in Pulses.ModelCull (i). Overlaid on the histograms are fits from a two-component Gaussian mixture model (black and red lines) and the two underlying Gaussian components (grey lines). The inset in i shows just the mixture model fits for both datasets. IPI: inter-pulse interval.

We developed software for segmenting song, called *FlySongSegmenter *(Figure 2), which, at its core, employs the continuous wavelet transform [17] to detect individual pulses and multitaper spectral analysis [18] to detect sine trains. We then compared the automated detection of pulse and sine events with a sample of manually annotated recordings (Figure 3). With optimized parameters for *D. melanogaster*, wavelet detection of song pulses combined with heuristic rules for winnowing (resulting in Pulses.IPICull) displayed high sensitivity (Figure 3a, b), but sometimes relatively low positive predictive values (Figure 3c), especially for recordings that contained non-song sounds (such as grooming and jumping). To reduce the number of false positives detected, we employed a likelihood-based approach to winnow putative pulses (resulting in Pulses.ModelCull; see Additional file 3). Likelihood-based winnowing improved the positive predictive values with minimal reduction in the sensitivity (Figure 3b, c). Because of the tradeoff between sensitivity and positive predictive value in these recordings, all of the automated outputs therefore had similar overall accuracy (Figure 3d). After culling with the likelihood model, we confirmed that the automated outputs produced estimates of the inter-pulse interval - a key song parameter - that were similar to those estimated from manually annotated song (Figure 3g, h).

The sine trains detected by multitaper spectral analysis were shorter (Student's *t *= 5.2, 8 degrees of freedom (df), *P *< 0.001) and more numerous (Student's *t *= -7.0, 8 df, *P *< 0.001; Figure 3e, f) than those scored by manual annotation. This confirmed that, although our sample of manually annotated data came from a relatively small sample of song, this sample was of sufficient size to detect systematic biases resulting from automated segmentation. The differences between sine train length and number may result either from over-splitting of sine trains by FlySongSegmenter or by over-grouping of sine trains during manual annotation (Additional file 3). However, the systematic biases balanced out such that FlySongSegmenter detected approximately the same amount of total sine song as manual annotation (Student's *t *= -1.5, 8 df, *P *= 0.16; Figure 3g). Thus, FlySongSegmenter demonstrates high overall accuracy for detecting both pulse and sine trains in fly song.

### Natural variation in song features

**Figure 4:**
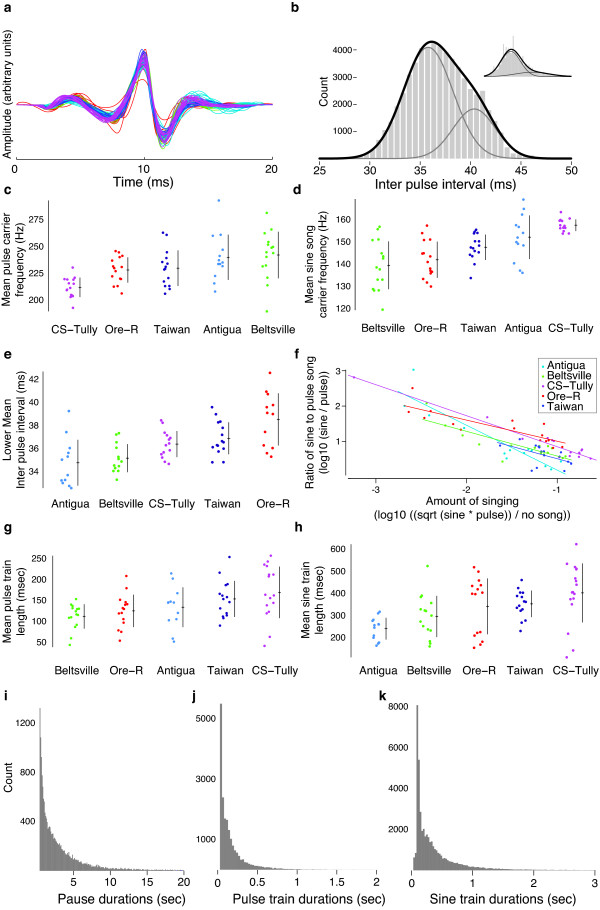
**Statistics of *D. melanogaster *song**. **(a) **Mean (per individual) re-scaled pulse shapes from five wild-type strains: Antigua = light blue; Beltsville = green; CS-Tully = purple; Oregon-R = red; Taiwan = dark blue. The same colors indicate strains in later panels. **(b) **Histogram of inter-pulse intervals over the entire dataset. Inset illustrates the distribution for one individual. A two-component Gaussian mixture model (dark line) and the two Gaussian components (light lines) are shown fitted to the data. **(c, d) **The average pulse (c) and sine train (d) carrier frequencies vary between individuals and amongst strains. **(e) **The mean inter-pulse interval, here shown for the lower component of a Gaussian mixture model, showed considerable variation amongst strains. **(f) **The relative amounts of sine and pulse trains were treated as a "three-way choice' - between sine train, pulse train, and no song - and plotted as recommended by Schilling *et al. *[45]. This method reveals that flies that produced more song overall sang proportionately fewer sine trains relative to pulse trains. In addition, strains displayed significant heterogeneity in the relative amounts of sine and pulse trains that they produced. **(g**,**h) **Both pulse train length (g) and sine train length (h) exhibit variation amongst strains. **(i**-**k) **The duration of all pauses between song bouts (i), the duration of pulse trains (j), and the duration of sine trains (k) each exhibit extensive variation.

We validated the utility of this platform by collecting and segmenting 14-minute recordings from each of 88 courting males sampled from five non-mutant, wild-type strains of *D. melanogaster*. In our recordings, we detected a majority of pulses with maximum energy at approximately 220 Hz (Figure 4a, c), the fundamental carrier frequency, as has been reported previously [19]. Approximately 17% of pulses had maximum energy at roughly double this frequency, likely reflecting the second harmonic (Figure S5 in Additional file 3).

We estimated models of pulse shape for each recording (Figure S5 in Additional file 3). Consistent pulse models could not be constructed for individuals that produced fewer than approximately 100 pulses and these individuals were thus excluded from further analysis. For the remaining individuals (N = 75), we detected an average of 2,418 pulses from each male (SD = 1,731; range, 182 to 7,879). All individuals displayed pulse models with similar shapes (Figure 4a), although the five strains displayed significant heterogeneity in pulse carrier frequency (Figure 4c; analysis of variance (ANOVA), F_4,68 _= 7.01, *P *< 1e^-4^).

Different strains also displayed heterogeneity in sine carrier frequency (Figure 4d; ANOVA, F_4,67 _= 11.35, *P *< 1e^-6^). We observed no significant correlation between the average carrier frequencies of pulse and sine trains, either among strains (compare Figure 4c and 4d), or across the entire dataset (Table S1 in Additional file 3).

Pulse trains are composed of a series of pulses that are separated by an IPI, a rapidly evolving component of song that is important for female choice [3,4,10,20]. The distribution of over 100,000 IPIs over the entire dataset is skewed to longer IPIs (Figure 4b), as has been noted previously [10,11]. We discovered that a two-component Gaussian mixture model fit the data better than most other simple models (Figure 4b). The mean of the lower Gaussian fit also displayed significant heterogeneity amongst strains (Figure 4e: ANOVA, F_4,61 _= 9.54, *P *< 1e^-5^).

### Temporal dynamics of courtship song

Song bouts often comprise alternating pulse and sine trains of various lengths (Figure 2 and Additional file 3). On average, flies that sang more song overall produced relatively less sine song (Figure 4f; for slope, T = -10.48, 73 df, *P *< 0.0001). Different strains produced different proportions of sine versus pulse trains (Figure 4f; analysis of covariance, for intercepts F_4,64 _= 9.11, *P *< 0.0001). We found heterogeneity among strains in both pulse train length (Figure 4g; ANOVA, F_4,68 _= 3.63, *P *< 0.01) and in sine train length (Figure 4h; ANOVA, F_4,70 _= 5.08, *P *= 0.001). Pulse and sine train lengths were weakly correlated across the entire dataset (*rho *= 0.47; Table S1 in Additional file 3).

We estimated all pauses between song bouts (consecutive collections of sine and pulse trains that were separated by less than 0.5 seconds) and found a wide distribution of pause durations (Figure 4i). We also examined the distributions of all pulse train and sine train durations and found that they, too, displayed broad distributions (Figure 4j, k).

**Figure 5:**
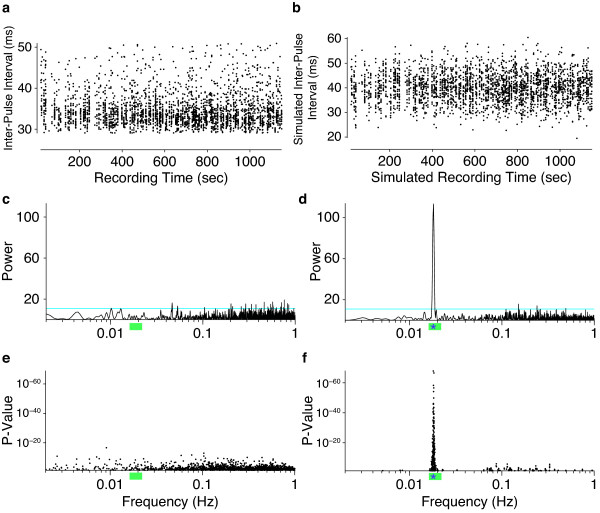
**Statistical analysis of rhythms in the inter-pulse interval**. **(a) **An example of inter-pulse intervals produced by a single individual during a single recording session. **(b) **An example of simulated inter-pulse intervals with a periodicity of 0.018 Hz, a SNR = 1, and sampling times derived from the data in panel a. (**c**,**d**) The Lomb-Scargle periodograms for the real data shown in panel a (c) and for the simulated data shown in panel b (d). Horizontal cyan lines indicate power where *P *= 0.05. **(e, f) ***P*-values of the local peaks in the Lomb-Scargle periodograms of inter-pulse interval for all 75 recordings of *D. melanogaster *(e) and for all 75 simulated datasets with a SNR = 1 (f) over the range of 0 to 1 Hz. Only *P*-values below 0.05 are plotted. The green bars below the axes in c-f mark the range of 0.016 to 0.22 Hz, which is the reported range of rhythms in the inter-pulse interval [9,21-23,46]. The asterisks within the green bars in d and f indicate 0.018 Hz, the frequency used in these simulations. The naturally skewed distribution of the real data, for example in panel a, differs from the distribution in the simulated data. We found, however, that culling the data to generate Gaussian distributed inter-pulse interval data resulted in even fewer significant peaks in the periodograms than shown in panel e and no obvious clustering of peaks in any particular frequency range (not shown). SNR: signal-to-noise ratio.

Previously, several studies have reported that the inter-pulse interval exhibits periodicity, with a period of approximately 55 seconds (or 0.0182 Hz) in *D. melanogaster *[9,21-23]. It appears that none of the previous datasets have been of sufficient size to reliably estimate rhythms on the scale of minutes [24]. In addition, no previous analysis has dealt adequately with the fact that the inter-pulse interval is sampled unevenly in time [24]. Our data contain multiple samples of a length sufficient to rigorously assess the presence of rhythms. To search for these rhythms, we used the Lomb-Scargle periodogram [25,26] (Figure 5), which was developed originally to study astronomical phenomena and is an accepted method for estimating the periodogram of unequally spaced time series [27].

All of the songs displayed significant rhythms in multiple frequency ranges (Figure 5c, e), but a minority of songs (29) displayed rhythms with power in the relevant frequency range of 0.016 to 0.022 HZ (*P *< 0.05; Figure 5e). For the songs that displayed significant power in the relevant range, frequencies outside of the relevant range usually displayed equal or stronger power (Figure 5c, e). There is, therefore, no compelling signal of periodicity limited to any particular frequency range for the inter-pulse interval data in this dataset. To determine whether this test was sufficiently powered to detect rhythms in the relevant range, were they to exist, we simulated inter-pulse interval rhythms of 0.0182 HZ with sampling times taken from our recorded songs, combined this rhythm with Gaussian noise of various levels (for example, Figure 5b), and then calculated the Lomb-Scargle periodograms for these simulated datasets (Figure 5d, f). Even when the simulated periodicity was buried in considerable noise, the Lomb-Scargle periodogram displayed excellent power to detect periodicity (Figure 5f). Over the signal-to-noise range of 0.1 to 2 (lower than the SNR for our recordings), our power to detect significant periodicity between 0.016 and 0.022 HZ exceeded 0.80 when the amplitude of the simulated periodic signal was at least equal to the variance in inter-pulse interval (SNR > 1; Figure S10 in Additional file 3).

**Figure 6:**
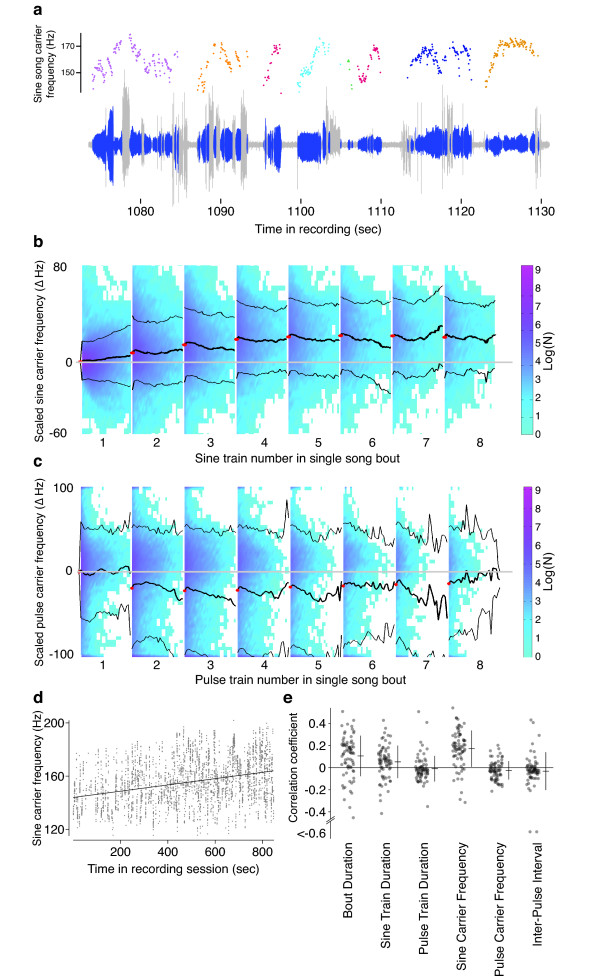
**Song dynamics in bouts**. **(a) **The carrier frequency of sine trains is modulated during song bouts. An example raw song trace is shown below in grey with sine trains shown in blue and the sine carrier frequency, measured in 50 ms bins, is shown as colored points above. Different colors indicate separate bouts of song. **(b) **Averaged across the entire dataset, the sine carrier frequency tends to increase over the course of a single bout. The frequency of sine trains within a bout was subtracted from the mean frequency of the initial bin of the first sine train. Histograms of the distribution of data at each time point are illustrated as colored plots across time, with a scale bar on the right indicating the log of the sample size. The mean and the standard deviation of the scaled carrier frequency are indicated with bold and thin black lines and the scaled carrier frequency at the beginning of each sine train within a bout is indicated with a red point. **(c) **The scaled average pulse carrier frequency displays a noisy decreasing trend over the course of a song bout. Colors and symbols as in b. **(d) **Sine train carrier frequency as a function of recording time for a single individual displays an increase in the mean carrier frequency with time. **(e) **The correlation coefficients for multiple song parameters as a function of recording time for all individuals for bout duration, sine train duration, pulse train duration, sine carrier frequency, pulse carrier frequency, and inter-pulse interval.

Although we did not detect previously reported long rhythms in the inter-pulse interval, we did find other significant patterns in song. For example, we noticed that the fundamental frequency of sine trains was highly variable and appeared to be modulated during song bouts (Figure 6a). This pattern of sine modulation has not been noted previously. We used the Lomb-Scargle periodogram to test for long time scale rhythms in sine train fundamental frequency and we found no evidence for consistent fluctuations on the order of seconds (Figure S7 in Additional file 3). Our simulations showed that our power exceeded 0.99 with a SNR of at least 0.1 to detect such rhythms (Figures S8 and S9 in Additional file 3).

We therefore examined whether sine train fundamental frequency varied in a consistent manner within song bouts. We found that sine carrier frequency increases over the first few sine trains within a song bout (Figure 6b), and that all five strains display this pattern (Figure S10 in Additional file 3). We tested whether this phenomenon arises from a general increase in motor activity during each song bout - as might be expected from the warming of an active fly - by examining the within-bout dynamics of pulse carrier frequencies. Consecutive pulse carrier frequencies are more variable than for sines and they do not display any general tendency to increase during a song bout (Figure 6c). If anything, pulse carrier frequency tends to decline during a song bout. Combined, these results suggest that male flies modulate sine fundamental frequency during a song bout and that this modulation is not driven by simple thermodynamic constraints.

We tested for trends across an entire recording by measuring the correlation between elapsed time during the recording and various song parameters. We found that the average sine train carrier frequency increased over the course of most recordings, by about 10 to 20 Hz (Figure 6d, e; t-test for equality of correlations on average with 0, *t *= 8.97, 74 df, *P *< 0.0001). In addition, we found a tendency for bout duration (t = 4.98, 74 df, *P *< 0.0001) and sine train duration (t = 3.05, 74 df, *P *< 0.005) to increase during recordings (Figure 6e). Previous studies have suggested that females do not use sine trains to guide mating decisions [28,29]. However, most previous studies have not used natural sine trains, and certainly not sine trains that were modulated as we have observed, in tests of female preference. Our observation of striking temporal dynamics of sine train carrier frequency, both within bouts on the order of seconds and over the course of an entire 14-minute courtship trial, suggests that it may be fruitful to reexamine the role of sine song in *Drosophila *courtship.

## Discussion

We have developed hardware and associated software that allows rapid collection and segmentation of an unprecedented amount of courtship song from *D. melanogaster*. We have illustrated the power of this system by analyzing a large sample of song from five wild-type strains. All song parameters that we have examined display intra-individual, inter-individual, and inter-strain variation. Almost all aspects of song display heterogeneity amongst strains. Because these five strains represent independent isolates from nature, and because all strains were treated identically in the laboratory, the variation between the strains most likely results from genetic differences between strains [30]. Additionally, separate song features were either weakly correlated or uncorrelated (Table S1 in Additional file 3), which suggests that these strains segregate for multiple genetic variants that can influence each song feature independently. This observation is consistent with other studies that have revealed that many loci contribute to song variation [13,31,32]. These observations are also consistent with the extreme diversity of courtship song found amongst species of the genus *Drosophila *[3,4,10].

Previous studies of courtship song have reported a periodic cycling of the inter-pulse interval and that the frequency of this cycling is influenced by mutations at the *period *locus [9,23]. Furthermore, this rhythm was reported to differ between closely related species, at least partly as a result of natural variation at the *period *locus [21,22]. The existence of these rhythms proved to be controversial and several groups reported that they were unable to detect the rhythm [33,34]. One hallmark of these previous studies is that they all involved datasets of a modest size, none suitable for estimation of rhythms with periods on the order of tens of seconds [24]. Furthermore, most studies have employed spectral analysis techniques that are not appropriate for time series data sampled at irregular intervals. This limitation of classical spectral analysis was overcome by the development of new approaches by Lomb and Scargle that allow efficient detection of rhythms in irregularly sampled time series data. In addition, we have generated datasets where most individuals produced far more than 300 sample points, which, it has been suggested, is the absolute minimum sample size required to estimate significant power within a particular spectral range [35]. We did not detect any evidence for the existence of periodic cycles of the inter-pulse interval at any frequency (Figure 5). Our simulations confirmed that our dataset was of sufficient size to find these rhythms if they existed. We must therefore conclude that flies singing in our apparatus do not produce periodic cycling of the inter-pulse interval and that this phenomenon is not available to us for further study.

Given our difficulty in detecting long-term trends in the inter-pulse interval, we were surprised to find several temporal modulations in sine train carrier frequency. First, male flies tend to increase the sine carrier frequency over the first few trains of a song bout, and this effect is specific to sine trains. Since we found no comparable trend for changes in pulse frequency, it appears that male flies modulate the sine carrier frequency independently of physical constraints on wing vibration. In addition, the independent modulation of sine train frequency and pulse frequency suggests that these two modes of song are produced by different mechanisms. These results contrast with the proposal of Ewing, who suggested that pulses are generated by a dampening of sine song [36,37].

We also discovered that male flies modulate sine carrier frequency over even longer time scales. Over the course of the entire recording session of 14 minutes, average sine fundamental frequency tended to increase in most recordings (Figure 6d, e). In addition, song bout duration and sine train duration also displayed linear trends over this time scale (Figure 6e). Our results indicate that fly song, and sine song in particular, contains patterns on multiple time scales. It remains to be seen how much of this variability is detected and utilized by females for mate discrimination.

We observed extensive variation in many of the temporal dynamics of song, such as the pauses between bouts and the lengths of bouts and trains. This temporal variability in the structure of courtship song contrasts sharply with the highly stereotyped calling songs of other insects, such as crickets and grasshoppers [38]. Currently, it is not clear how the variability in *Drosophila *courtship song is generated at the neural level. Male flies may switch between two central pattern generators to produce sine and pulse trains, a mechanism suggested by the apparently independent genetic variation for sine and pulse parameters, but we do not know what determines the length of time spent in each mode. We suspect that the patterns of sine and pulse trains may be related to the dynamics of courtship behavior, and in particular to the interaction of the male and the female, as has been suggested recently [39].

New transgenic tools available in *D. melanogaster *provide the opportunity to manipulate sparse subsets of neurons to interrogate neural networks [40]. Our platform provides the opportunity to perform a systematic analysis of the neural basis for courtship song, even for neurons that contribute only subtle, quantitative aspects to song. In preliminary studies, we have found that our hardware and software can be employed, with minor modifications, to record and analyze song from several other species of *Drosophila*. We expect, therefore, that our platform will accelerate research on the genetic and neural basis for song within *D. melanogaster *and also will aid studies on song evolution.

## Methods

### Biological materials

Five strains of *D. melanogaster *were studied: Antigua = W-22 Antigua 89 from M. Ashburner; Beltsville = Beltsville, Missouri from M. Ashburner; CantonS (T. Tully); Ore-R = W-38 Oregon-R from M. Ashburner; and Taiwan = 14021-0231.07 from the University of California at San Diego stock center. Virgin males were collected and aged for 4 to 6 days. Virgin females at least one day old with their arista removed were used for recording with virgin males. Recordings were performed at approximately 25°C and 50% relative humidity.

### Software validation

One minute of song (from minutes 5 to 6 of each recording) was hand-annotated by TS-M using custom MatLab software (*FlySongSegmenterByHand.m*) that is part of the *FlySongSegmenter *suite. We calculated the sensitivity (number of hand-annotated pulses found by *FlySongSegmenter*/number of annotated pulses) and positive predictive value (number of hand-annotated pulses found by *FlySongSegmenter*/number of pulses found by *FlySongSegmenter*). We also calculated F, the harmonic mean of the sensitivity and positive predictive value (F = (2 × sensitivity × positive predictive value)/(sensitivity + positive predictive value)), where F = 1 implies perfect classification.

### Statistical analyses

All statistical analyses were performed in MatLab R2011b. Differences in sine statistics between segmented and hand-annotated song were tested using paired, two-tailed Student's *t *tests. Statistical outliers were identified and removed prior to statistical analysis and plotting with an iterative Grubb's test [41,42]. Heterogeneity amongst strains in most song parameters was tested using a one-way ANOVA and for the relative amount of sine to pulse song compared to total song using analysis of covariance.

### Additional methods

Additional information, including complete plans and instructions for building the hardware, using the software, and further details of data analysis, are provided in Additional files 1, 2, 3, 4, 5, and 6.

### Data and software availability

The raw data from all recordings analyzed in the paper are available as .wav files [43] and the open-source MatLab software suite *FlySongSegmenter *is freely available [44].

## Abbreviations

ANOVA: analysis of variance; df: degrees of freedom; SD: standard deviation; SNR: signal-to-noise ratio.

## Competing interests

The authors declare that they have no competing interests.

## Authors' contributions

The project was conceived by DLS and MM. The electronics and data acquisition software were designed and developed by BJA. The recording chambers were developed primarily by TS-M. The data were collected and hand-annotated by TS-M. Analysis software was written by BJA, PC, DLS and MM. The data were analyzed by DLS and MM and the manuscript was written by DLS and MM, with input from all of the authors. All authors have read and approved the manuscript for publication.

## Supplementary Material

Additional file 1 **Parts and instructions for soldering the 32-channel amplifier for the courtship song recording system**. Detailed parts list and instructions for building and testing the amplifier described in the paper. Click here for file

Additional file 2 **Prior art and parts and settings used for new courtship chambers**. Table that lists microphones and amplifiers used previously in *Drosophila *courtship recording apparatuses and two tables that list parts and laser settings required to build the courtship chambers described in the paper. Click here for file

Additional file 3 **Additional methods and additional results**. Description of microphone performance, circuit design, acrylic courtship chambers, details of pulse and sine detection algorithms, and additional results. Click here for file

Additional file 4 **Testing and data acquisition software**. MatLab scripts that allow testing the amplifier board during construction and data collection of the finished apparatus. Click here for file

Additional file 5 **Acrylic cdr files**. CorelDRAW files for the laser cutter to cut all parts used in the apparatus. Click here for file

Additional file 6 **Amplifier pcb and sch files**. pcb and sch files that provide design and detail for custom amplifier board. These files can be used to order printed circuit boards for the 32-channel amplifier board. Click here for file
