# Supplementary material for: Multi-channel acoustic recording and automated analysis of Drosophila courtship songs
Source: BMC Biol. 2013 Jan 31;11:11. doi: 10.1186/1741-7007-11-11 (PMC3599446; doi:10.1186/1741-7007-11-11)
Supplement: Additional file 1 — Parts and instructions for soldering the 32-channel amplifier for the courtship song recording system. Detailed parts list and instructions for building and testing the amplifier described in the paper. [file 1741-7007-11-11-S1.pdf]

Additional file 1

Parts and instructions for soldering the 32-channel amplifier for the courtship song recording system.

INTRODUCTION

These are assembly instructions for building a 32-channel amplifier for the courtship song recording system described in Arthur et al.(2013). Similar procedures apply to the 2-channel version, which has BNC, 3.5-mm phono, and terminal block outputs, instead of mass termination pin receptacles.

REQUIREMENTS

You will need a NI-USB-6259 digitizer, a newish MS-Windows computer, and 32-bit Matlab with the Data Acquisition Toolbox. The 2-channel version can be hooked up to the audio input jack on any computer (e.g. a laptop for field use).

Note that 64-bit Matlab (as of release 2011b) won't work because the digital I/O used by the hygrometer works only for NI products with the 32-bit DAQ toolbox. 32-bit Matlab can be installed on 64-bit MS-Windows.

FILES (These are provided along with these instructions.)

amp32.sch is a schematic of the circuit. *amp32.pcb* is the layout. Both are readable by free MS-windows software from expresspcb.com. You can edit *amp32.sch* to update the parts list below with custom gain and filter settings. Print out a new parts list by pulling down the Edit menu. To order boards, open *amp32.pcb* and pull down the Layout menu. *amp32.sch* and *amp32.pcb* should look like the following screen shots (if they do not, contact the authors for the correct files).

amp32.sch

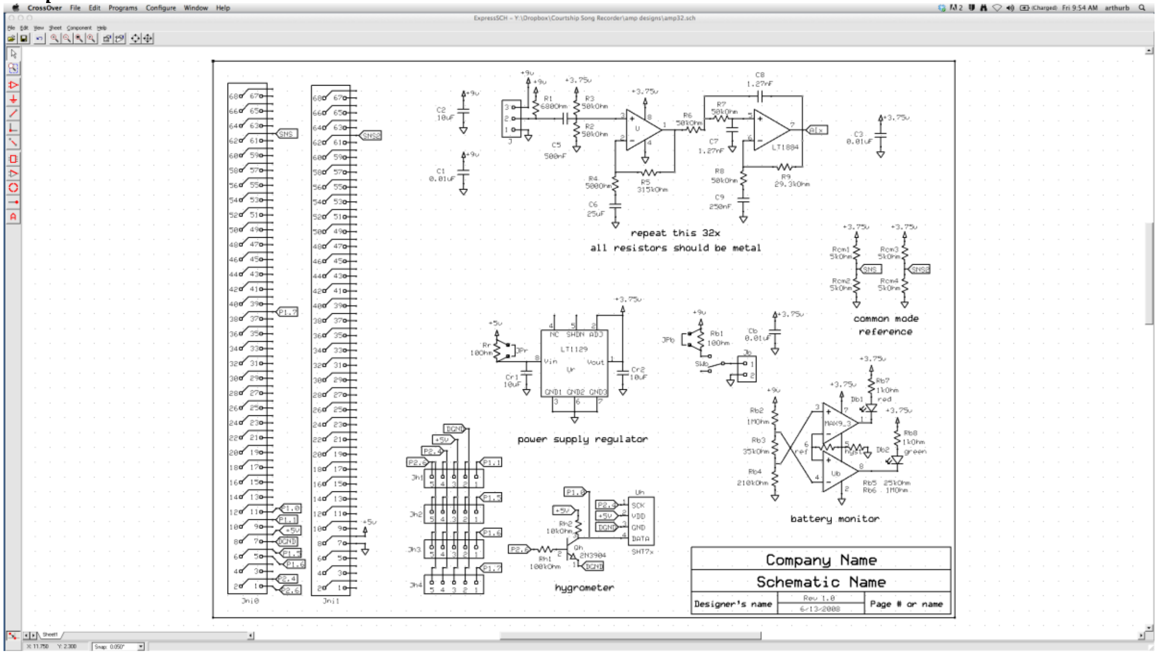

amp32.pcb

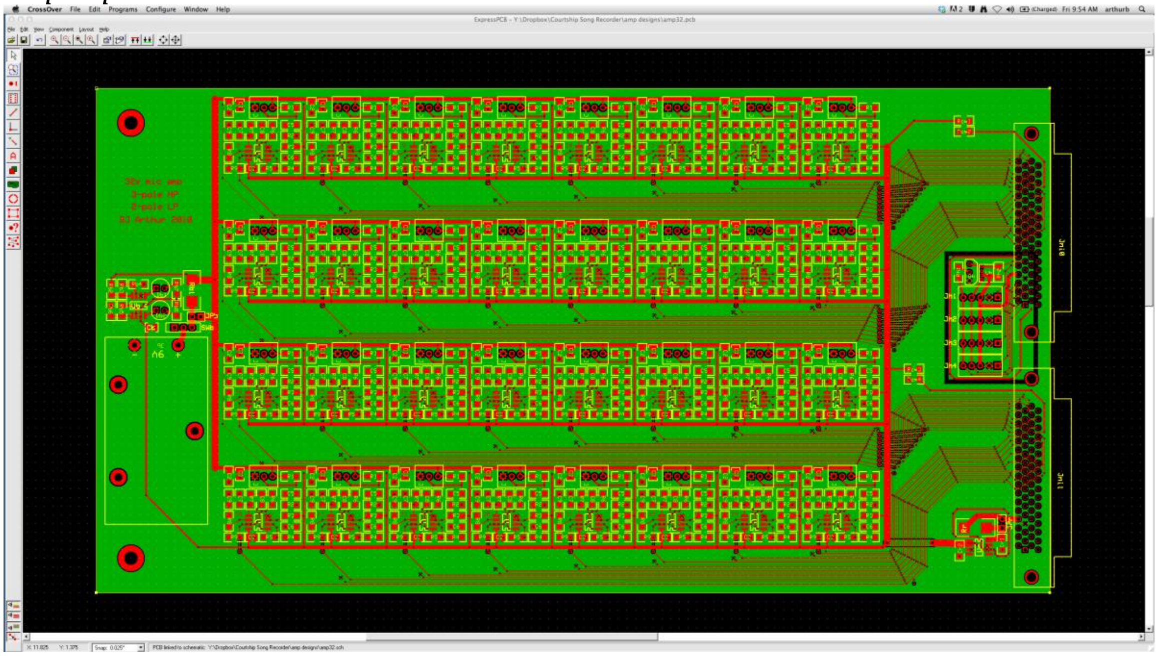

amp32h.pcb contains additional (and optional) hygrometer daughter boards. The main board includes space for one hygrometer, plus jacks for four remote boards. *amp2.pcb* (sic, not *amp32.pcb*) contains four hygrometers daughter boards in the layout, which can be removed from the board with a band saw.

array_take.m is Matlab software used to acquire 32-channel data plus hygrometer data from the *amp32.pcb* board via a NI-USB-6259 digitizer. Note, you can also simultaneously acquire video data with array_take.m from any camera connected to the USB port of the computer. You may have to do some work to synchronize the audio and video signals.

freq_resp_take.m, *freq_resp_plot.m*, and *crosstalk_fig.m* are Matlab software used to test the transfer function and noise characteristics of the board after it's built.

PARTS

All part numbers below are from digikey.com circa 2011.

All resistors should be metal (or thin film) to minimize noise.

Gain of the amplifier is equal to R5/R4*1.59, and can be adjusted via R5. Our layout employs a gain of ~1000X, which is appropriate for small (i.e. quiet) flies like *D. melanogaster*. The gain can be reduced if you will be recording from larger, louder flies. Alternatively, you can just place spacers between the microphone and courtship chamber to record from larger flies.

Keep R2=R3 and C5*R2/2 = C6*R4 = C9*R8. High-pass cutoff is 1.96/(2*pi*C6*R4), and can be adjusted via the capacitors.

Keep R6=R7 and C7=C8. Low-pass cutoff is 1/(2*pi*C7*R6), and can be adjusted via the capacitors.

The product of the gain and the low-pass cutoff should not exceed the gain-bandwidth product specification of the op-amp, which is 5 GHz for OPA376.

Two different microphones can be used: Knowles' NR-23158 or CUI's CMP-5247.

Omit R1 for the Knowles NR-23158 microphone. Use R1=680 Ohm for the CMP-5247 microphone.

Two different op-amps can be used for Ur: OPA376 and LT1884. Take your pick.

For each channel:

**Board part identified**	**Type**	**Digi-key part #**
C1,C3,Cb	0.01 uF Capacitor	490-4339-1-ND
C2	10 uF Capacitor	445-1601-1-ND

C5	500 nF Capacitor	445-4032-1-ND
C6	25 uF Capacitor	490-4739-1-ND
C7,C8	1.27 nF Capacitor	478-1531-1-ND
C9	250 nF Capacitor	478-1561-1-ND
J	Connectors	WM2001-ND, WM4201-ND, WM2756-ND
R1	680 Ohm Resistor	RG32P680BCT-ND
R2	50 kOhm Resistor	RG32P49.9KBCT-ND
R3,R6,R7,R8	50 kOhm Resistor	RG32P49.9KBCT-ND
R4	500 Ohm Resistor	RG32P499BCT-ND
R5	315 kOhm Resistor	RG32P300KBCT-ND
R9	29.3 kOhm Resistor	RG32P30.0KBCT-ND
U	LT1884 Amplifier	lt1884cs8#pbf-nd

For each board:

**Board part identified**	**Type**	**Digi-key part #**
Cr1,Cr2	10 uF Capacitor	445-1601-1-ND
Db1	LED – red	516-1325-ND
Db2	LED - green	516-1327-ND
Jb	9V battery holder	BH9V-PC-ND
Jh1,Jh2,Jh3,Jh4	Connector header	WM4203-ND
Jh1,Jh2,Jh3,Jh4	Connector housing	WM2615-ND
Jni0,Jni1	Connector	A31808-ND
JPb,JPr	Shunt	609-3469-ND, A26228-ND
Qh	Transistor	2N3904FS-ND
Rb1,Rr	10 Ohm Resistor	PT9.1YCT-ND
Rb2,Rb6	1 MOhm Resistor	P1.0MECT-ND
Rb3	35 kOhm Resistor	311-34.8KFRCT-ND
Rb4	210 kOhm Resistor	RHM210KFCT-ND
Rb5	25 kOhm Resistor	RHM24.9KFCT-ND
Rb7,Rb8	1 kOhm Resistor	RMCF1/81KJRCT-ND
Rcm1,Rcm2,Rcm3,Rcm4	5 kOhm Resistor	TNP4.99KACCT-ND
Rh1	100 kOhm Resistor	541-100KECT-ND
Rh2	10 kOhm Resistor	541-10KECT-ND
SWb	Switch	679-1860-ND
Ub	IC Comparator	MAX983CSA+-ND
Uh	SHT7x Humidity Sensor	18m2988 (from newark.com)
Ur	Voltage regulator	LT1129CS8#PBF-ND

Tone-arm cable (Belden 8429: 2-conductor 7x40 gauge with braided shield) for microphone leads. Good luck finding it. Try eBay. Daburn 2746

(http://www.daburn.com/2741-2751Hi-FiStereoInterconnectingCable-PhonoWire.aspx) will also work.

PROCEDURE

VHDCI connectors: Insert Jni0 and Jni1 into the NI-USB-6259, position PC board on them, and solder in place; check for +5V between pins 7 and 8 on Jni1.

Voltage regulator: Solder up Ur, Rr, Cr1, Cr2, and JPr. Take care that Ur is oriented correctly: pin VIN should face Jni1; pin OUT should face away. Check for +3.75V on output. It should look like this approximately:

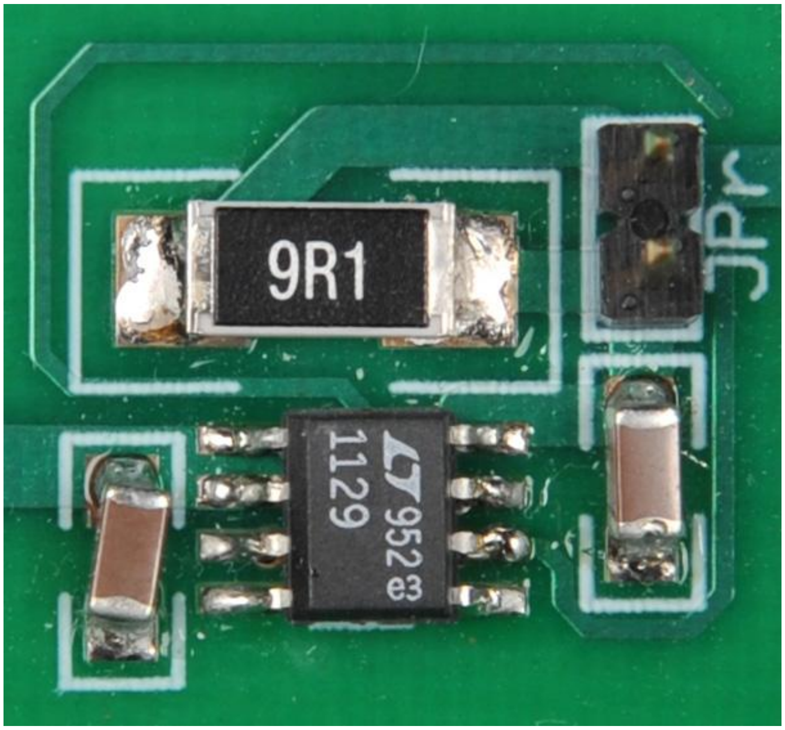

First channel: Solder up Rcm3, Rcm4, and channel 16 except R1. Pin V- of U should be over the through-hole in the board. Position the jack (WM4201) such that the plug (WM2001), when engaged, does not touch R7, C8, or R6. It should look like this:

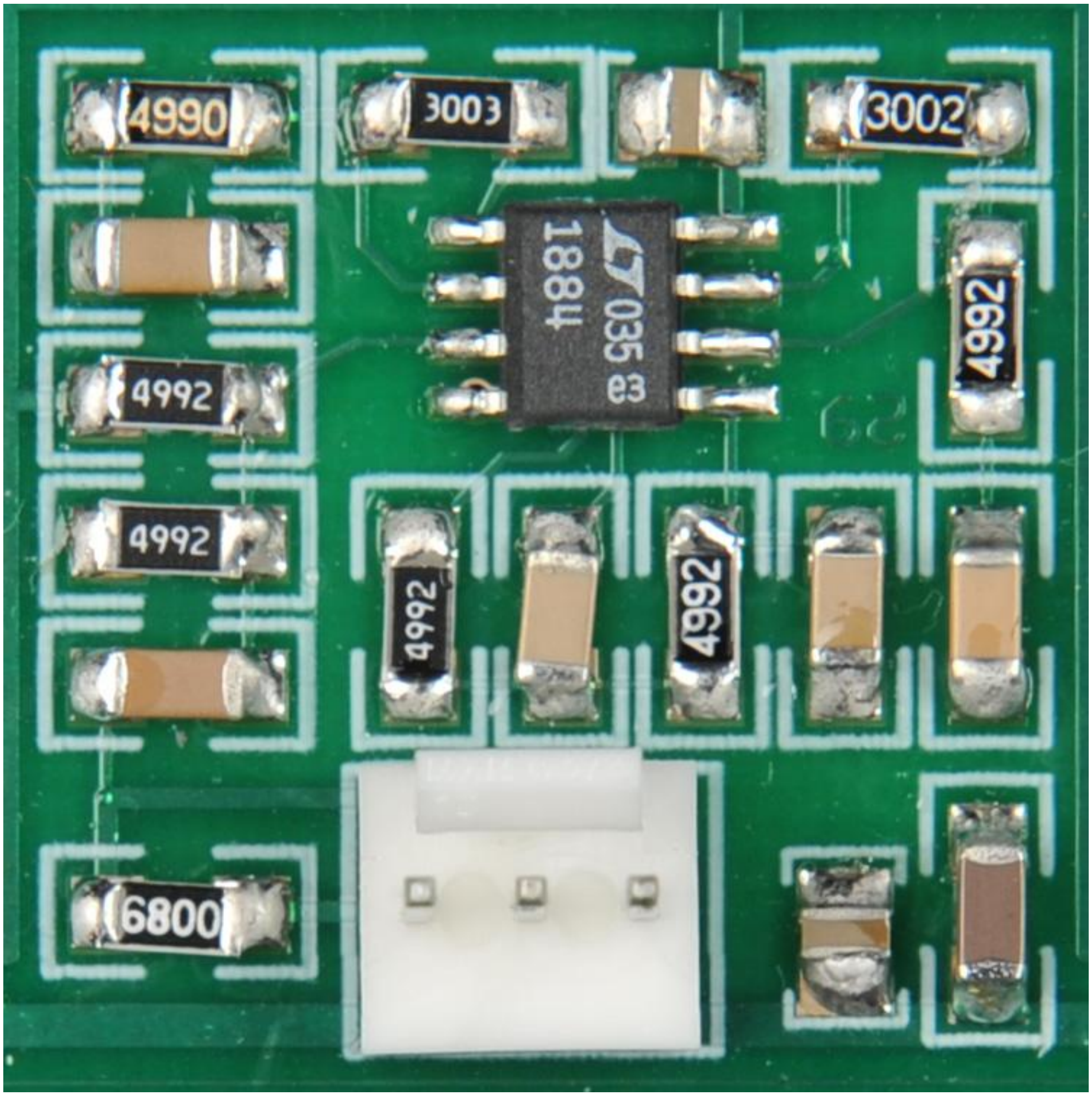

Create a voltage attenuator as follows: temporarily solder two bare wires to pins 21 (analog output 3) and 55 (ground) of Jni1. Connect the other ends to the top and bottom, respectively, of a resistive divider consisting of 100 kOhm or 1 MOhm on top, and 10 Ohm or 100 Ohm on the bottom, respectively. Test that a 10V input into the divider results in no more than a 1 mV output. Use two more bare wires to connect the output of the divider (the node between the two resistors) to the middle pin of a spare plug (WM2001) and the bottom of the divider to the plug's pin that connects to the square through-hole in the board. Like this:

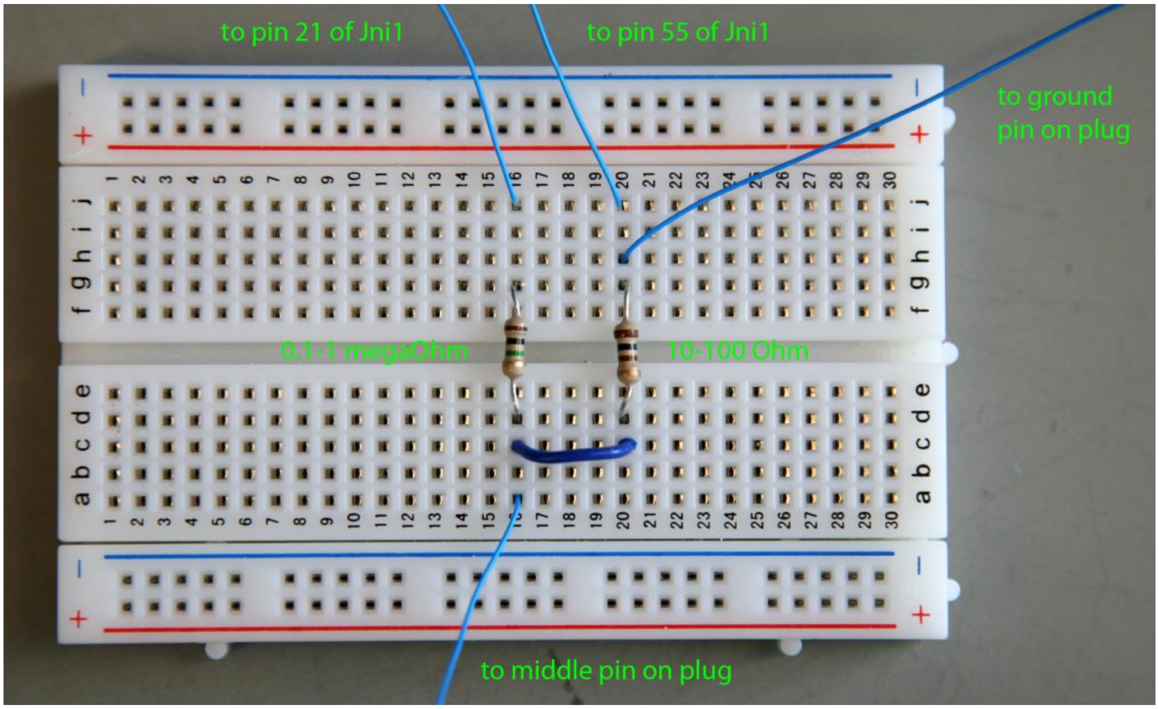

Synchronize the triggering of the analog output to the analog input by temporarily soldering pins 43 and 45 on Jni0 (PFI2 to PFI10) with a short piece of small gauge wire. If you're using a different NI-DAQ board that doesn't have RTSI, see http://www.mathworks.com/help/toolbox/daq/brty0qx.html. On the NI-USB-6211 you would screw PFI3 to PFI7. Like this:

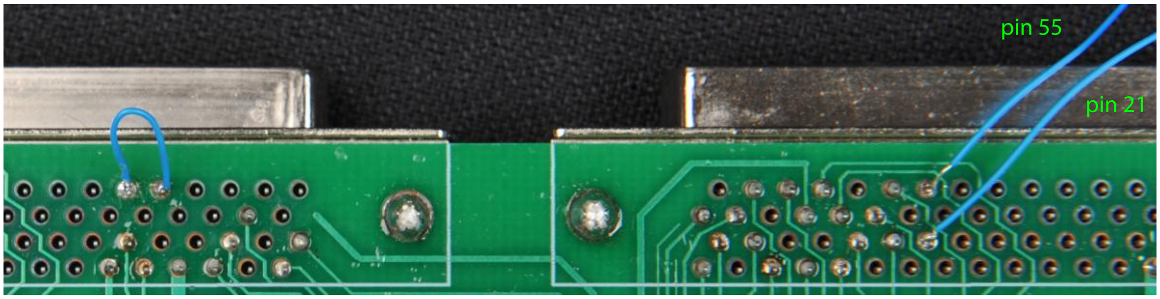

Test the frequency response and noise of channel 16 by executing the following command in Matlab:

```
>> freq_resp_take('chan16',logspace(1,4,25),3,16,10,80,1);
```

It should look like this:

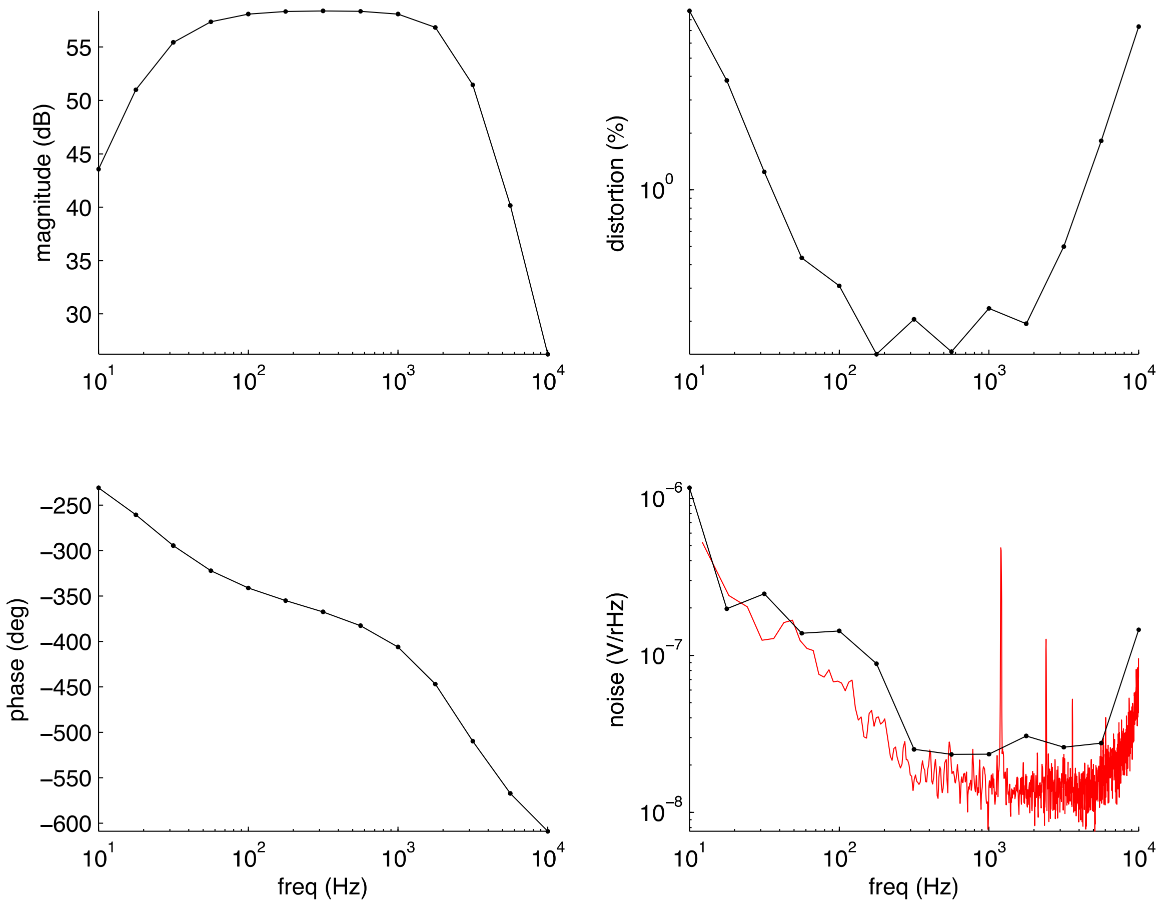

Check that the gain and cutoff frequencies are as you specified, and that the distortion and noise floor are < 1% and < 100 nV/rHz respectively.

Second channel: Solder up channel 25 except R1 and test as above:

```
>> freq_resp_take('chan25',logspace(1,4,25),3,25,10,80,1);
```

Check the crosstalk by leaving the test cable connected to channel 25 and connecting the channel 16 input jack to ground using a shunt (A26228):

```
>> freq_resp_take('chan16_cross',logspace(1,4,25),3,16,5,80,100);
>> crosstalk_fig('chan16','chan16_cross');
```

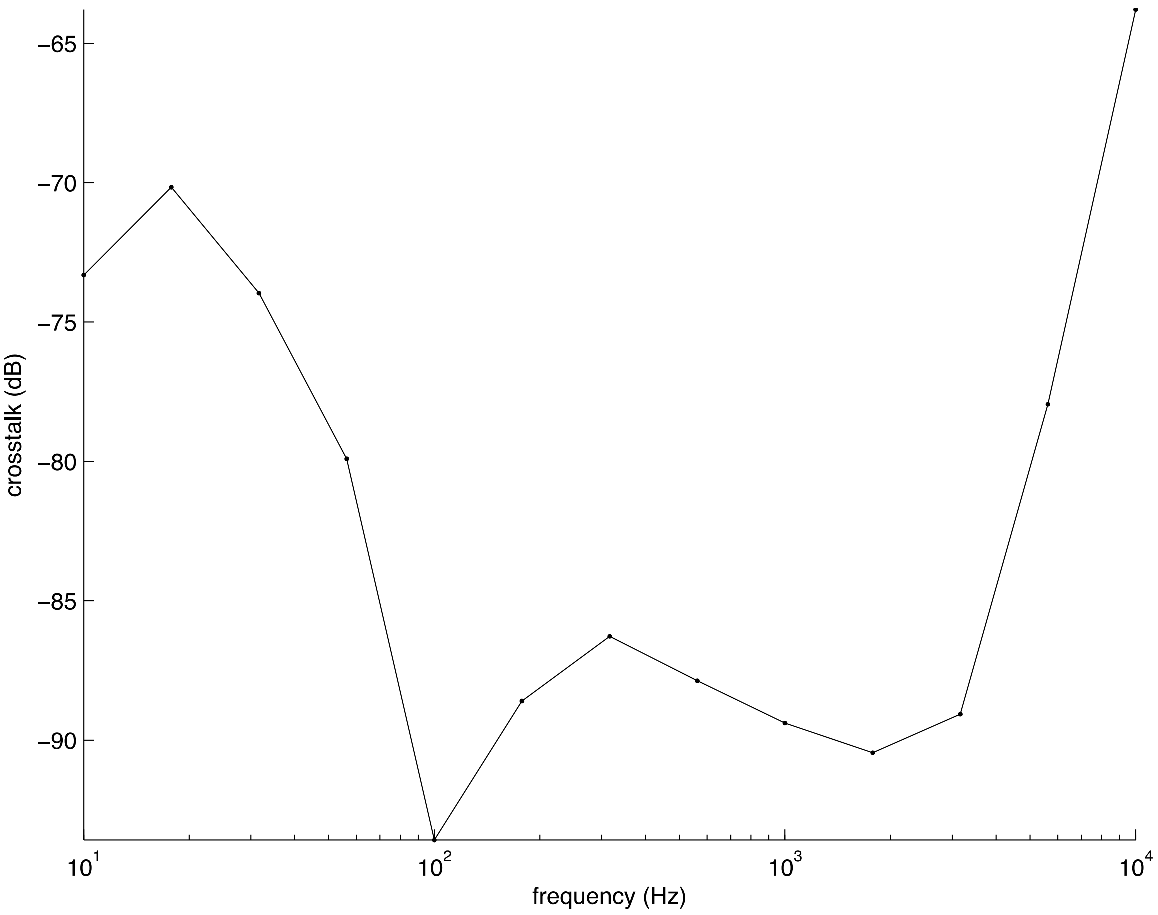

Crosstalk should be < -60 dB.

Microphones: Solder Jb, SWb, JPb and, if using the CMP-5247 mic, R1 on channels 16 and 25. Solder a microphone to a cable and a plug (WM2001). It is usually useful to prime the wires connected to the mics with a tiny bit of flux and a tiny bit of solder. One trick for soldering the plugs to the wires is to first partially insert the correct number of WM2756 pieces into a WM2001 (in the correct locations, see below) and tape the WM2001 to the counter. With the WM2756s "hanging out" from the WM2001, prime them with a bit of solder, then solder the wires. Finally, push the WM2756 home.

The negative lead on both mics goes to the pin that connects to the square through-hole in the board. Positive lead on CMP-5247 goes to the middle pin on plug. On the NR-23158 the output signal goes to the plug's middle pin, and the positive lead goes to the plug's other pin.

If necessary, configure the software to your preferences by editing the hardware settings found in array_take.m. Test that it works:

>>array_take

Once the GUI window appears, select channel 17 or 26 (add 1 to the board number b/c Matlab is unit-offset) using the radio buttons on the bottom, turn on the time series and spectrogram using the radio buttons on the right, click the green Start button in the upper right, and whistle into the mic and watch the window!

Hygrometer: Wire up either (1) Uh, Qh, Rh1, and Rh2 or (2) Jh1 and all the parts on amp32h.pcb. In both cases the black "chip" on Uh (SHT7) should face away from Qh, and the flat face of Qh (2N3904) should face Uh. For the latter, solder wires (stranded better than solid) from the 5 holes on amp32b.pcb to a jack (WM2615) and connect the jack to Jh1.

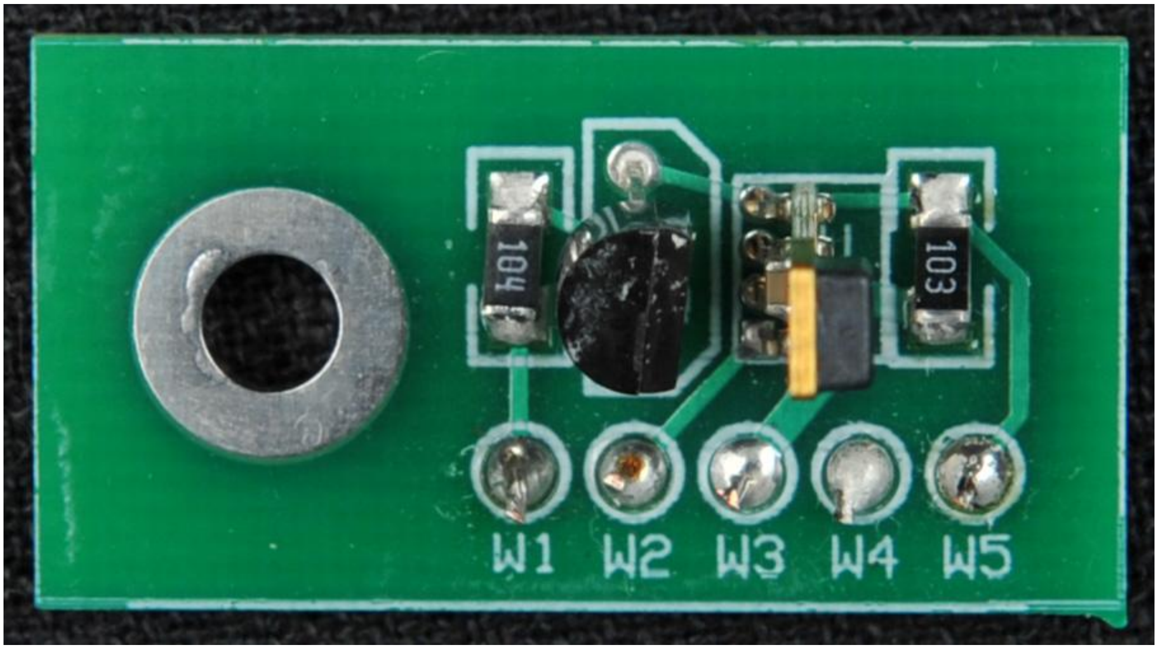

Test for accurate temperature and humidity readings:

>>array_take

Press the Start button and wait for temperature and humidity to be displayed in the upper left.

Battery monitor: Solder up Ub, Db1, Db2, Cb, and Rb2-8. The flat side on the LEDs should face the edge of the board. Pin 7 (V+) of Ub should face the battery. Insert and remove the battery to test that the green and red LEDs turn on correctly. Green

means fully charged; Green & Red together mean somewhat depleted; Red alone means replace with a new battery; no light means it's soldered up incorrectly.

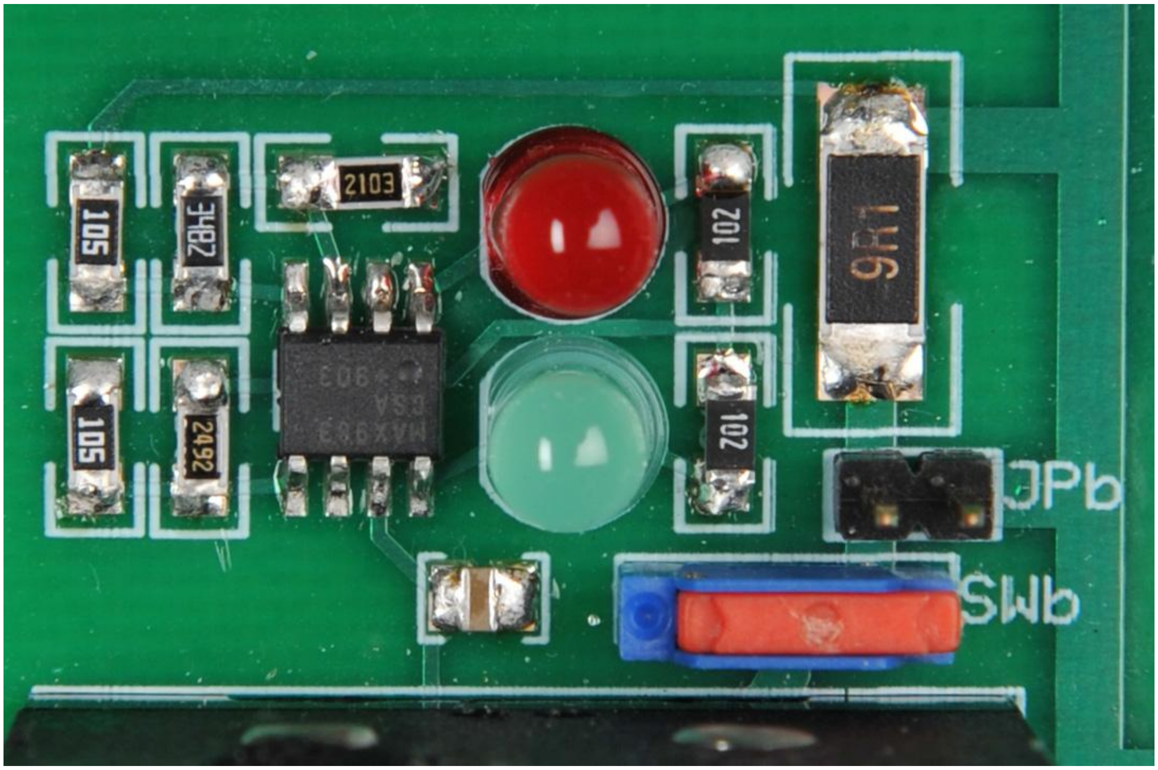

Remaining circuits: Solder up Rcm1, Rcm2, the remaining microphone channels, and the remaining hygrometers. Intermittently check that power has not been shorted to ground by measuring the resistance across C1 and C3. With no shunts across the microphone jack terminals, it should be at least a few megaOhm.

DEBUGGING

Normally JPb and JPr should be shunted. To measure the current drawn by the mics (and hence test for a short), remove the jumper from JPb, measure the voltage across the pins, and use Ohm's law with the value of Rb1 (9.1 Ohm). The current drawn by the amplifiers can be similarly measured across JPr.
