# Supplementary material for: Multi-channel acoustic recording and automated analysis of Drosophila courtship songs
Source: BMC Biol. 2013 Jan 31;11:11. doi: 10.1186/1741-7007-11-11 (PMC3599446; doi:10.1186/1741-7007-11-11)
Supplement: Additional file 2 — Prior art and parts and settings used for new courtship chambers. Table that lists microphones and amplifiers used previously in Drosophila courtship recording apparatuses and two tables that list parts and laser settings required to build the courtship chambers described in the paper. [file 1741-7007-11-11-S2.pdf]

Additional file 2

Title: Prior art and parts and settings used for new courtship chambers

Additional Table S2. Publications that first reported a new microphone or amplifier design for recording Drosophila melanogaster courtship song, along with several key features of each design.

| Publication | Microphone | Amplifier | Bandpass filter | Chamber size |
|---|---|---|---|---|
| (Shorey 1962) | Altex 21 D condenser | ND | ND | 12 mm D x 10 mm H |
| (Ewing and Bennet-Clark 1968) | Acos 43-1 crystal mic | Low noise preamplifier | 10-1500 Hz | 40mm D x 5mm H |
| (Bennet-Clark 1972) – Illustrated at bottom of table | Grampian ribbon mic | Low impedance amplifier, with design (200X) | 50-5000 Hz | |
| (Kyriacou and Hall 1980) | Reslo ribbon mic | Tandberg 10X reel-to-reel tape recorder | ND | 20x10x3mm |
| (Kawanashi and Watanabe 1980) | Dynamic mic | | | 10mm D x 3mm H, |
| (Crossley and McDonald 1980) | Brüel and Kjaer Type 4145 in sound attenuated room | | 120-750 Hz | 21mm D x 7mm H |
| (Cowling and Burnet 1981) | Reslo ribbon mic | Tandberg 3600 XD tape recorder | 50-500 Hz | 30x10x15mm |
| (Gorczyca and Hall 1987) | Insectavox – modified condenser microphone (Radio Shack #270-090) | LM387 | 80-1500 Hz + Notch filter (to reduce amplitude of sine song!) | |
| (Aubin, Rybak et al. 2000) | dual B&K 4176 6cm apart | | | 10mm D x 5mm H |

| | | | | |
|---|---|---|---|---|
| (Tauber and Eberl 2001) | Knowles Acoustics NR-23158 in wall of chamber | 741 opamp | | 9mm D x 6mm H |
| (Clyne and Miesenbock 2008) | Knowles Acoustics NR-23160 | Analog Devices AD620 | 10,000x | 10mm D |
| (Turner and Miller 2012) 10 recordings in parallel | GRAS Sound and Vibration 40PH array microphone | | <1k Hz | |

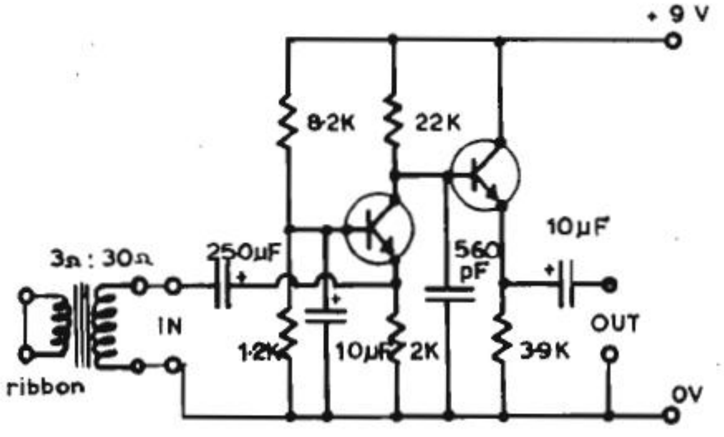


fig.2. LOW IMPEDANCE PRE-AMPLIFIER

**References**


**Aubin, T., F. Rybak, et al. (2000). "A simple method for recording low-amplitude sounds. Application to the study of the courtship song of the fruit fly *Drosophila melanogaster*." Bioacoustics 11: 51-67.**

**Bennet-Clark, H. C. (1972). "Microphone and pre-amplifier for recording courtship song of *Drosophila*." Drosophila Information Service 49: 127-128.**

**Clyne, J. D. and G. Miesenbock (2008). "Sex-specific control and tuning of the pattern generator for courtship song in Drosophila." Cell 133(2): 354-363.**

**Cowling, D. E. and B. Burnet (1981). "Courtship songs and genetic control of their acoustic characteristics in sibling species of the Drosophila melanogaster subgroup." Anim. Behav. 29: 924-935.**

**Crossley, S. and J. McDonald (1980). "A method for permanently recording courtship song and courtship behavior simultaneously in Drosophila." Drosophila Information Service 55: 150-151.**

**Ewing, A. W. and H. C. Bennet-Clark (1968). "The courtship songs of *Drosophila*." Behaviour 31: 288-301.**

Gorczyca, M. and J. C. Hall (1987). "The INSECTAVOX, an integrated device for recording and amplifying courtship songs." Drosophila Information Service 66: 157-160.

Kawanashi, M. and T. K. Watanabe (1980). "Genetic variation of courtship song of Drosophila melanogaster and D. simulans." Japan. J. Genetics 55: 235-240.

Kyriacou, C. P. and J. C. Hall (1980). "Circadian rhythm mutation in Drosophila melanogaster affect short-term fluctuation in the male's courtship song." PNAS 77: 6729-6733.

Shorey, H. H. (1962). "Nature of the Sound Produced by Drosophila melanogaster during Courtship." Science 137(3531): 677-678.

Tauber, E. and D. F. Eberl (2001). "Song production in auditory mutants of Drosophila: the role of sensory feedback." Journal of comparative physiology. A, Sensory, neural, and behavioral physiology 187(5): 341-348.

Turner, T. L. and P. M. Miller (2012). "Investigating Natural Variation in Drosophila Courtship Song by the Evolve and Resequence Approach." Genetics.

Additional Table S3. List of parts used in construction of courtship chambers, acrylic platform and acrylic shielding.

| | Parts | Vendor | Cat. No. | Item description |
|---|---|---|---|---|
| Chambers | Chamber top | McMaster-Carr | 8490K182 | 2 mm thick transparent acrylic |
| | Chamber bottom | McMaster-Carr | 8560K192 | 3 mm thick transparent acrylic |
| | Screw for a chamber | McMaster-Carr | B000FN68D0 | Nylon 6/6 Thumb Screw, Knurled Head |
| | Chamber divider | | | 0.25mm stainless steel |
| | Nylon mesh | Small Parts Inc. | B000FMWH6I | Nylon 6 Woven Mesh Sheet, Square Openings 350um |
| Acrylic platform | Microphone mounting table | McMaster-Carr | 8560K219 | 4.5 mm thick transparent acrylic |
| | Mounting standoff | McMaster-Carr | 93620A199 | Male-Female Thread Hex Standoff 1/2" Hex, 2" Length, 1/4"-20 Screw Size |
| | | | 93505A183 | Aluminum Male-Female Threaded Hex Standoff 1/2" Hex, 1/2" Length, 10-32 Screw Size |
| | Breadboard | Thorlabs | MB1824 | Aluminum Breadboard, 18" x 24" x 1/2", 1/4"-20 Threaded |
| | Vibration absorbing feet | Thorlabs | AV1 | 1/4"-20 Thread |
| Acrylic shielding box | Acrylic shielding box | McMaster-Carr | 8560K243 | 12 mm thick transparent acrylic |
| | Epoxy | Amazon | B000ALJ4NS | Epoxy for General Purpose |
| | Foam tape | Amazon | B000CSIBCO | High Density Foam Tape |
| | Clay | Dick Blick | 33268-2521 | Modeling Clay |

Additional Table S4. Settings applied to cut acrylic on UNIVERSAL Laser Systems PLS4.75 laser cutter.

**chamber-bottom**

| line color | power | speed | PPI |
|---|---|---|---|
| red | 100% | 5% | 500 |
| black | 100% | 14% | 1000 |
| blue | 100% | 5% | 500 |

image density=6

**chamber-top**

| line color | power | speed | PPI |
|---|---|---|---|
| red | 100% | 7% | 500 |
| black | 100% | 90% | 500 |

density=1

**microphone mounting table**

| line color | power | speed | PPI |
|---|---|---|---|
| red | 100% | 2.5% | 500 |
| black | 100% | 90% | 500 |

density=6

**sound shielding box**

| line color | power | speed | PPI |
|---|---|---|---|
| red | 100% | 1% | 500 |
| black | 100% | 90% | 500 |

density=1

**nylon mesh**

| line color | power | speed | PPI |
|---|---|---|---|
| red | 100% | 80% | 500 |
