# Supplementary material for: Multi-channel acoustic recording and automated analysis of Drosophila courtship songs
Source: BMC Biol. 2013 Jan 31;11:11. doi: 10.1186/1741-7007-11-11 (PMC3599446; doi:10.1186/1741-7007-11-11)
Supplement: Additional file 3 — Additional methods and additional results. Description of microphone performance, circuit design, acrylic courtship chambers, details of pulse and sine detection algorithms, and additional results. [file 1741-7007-11-11-S3.pdf]

**Additional file 3**

## Hardware

### Amplifier

We tested four microphones that exhibited the best tradeoff between sensitivity and physical size: an original Bennet-Clark microphone modified from Radio Shack 270-090[1] that was provided by Ron Hoy; the Knowles NR-23158[2]; the CUI Inc. CMP-5247TF-K; and the PNM-3546L-R. The SNR for all four microphones is shown in Fig. S1. Two of these microphones exhibited particularly good signal-to-noise ratios: the NR-23158 and the CMP-5247K. The amplifier was designed to accommodate either of these microphones by the inclusion/exclusion of a single resistor in the circuit.

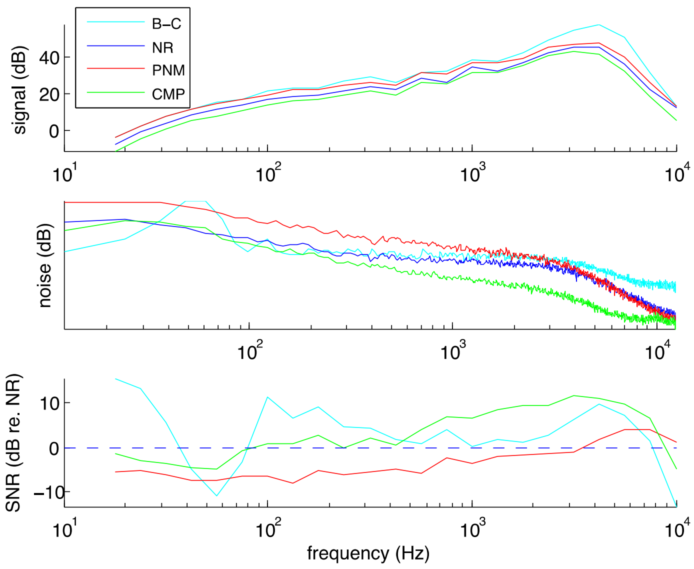


Fig. S1. Signal (top), noise (middle) and SNR relative to the NR-23158 (bottom) over the frequency range of 10 Hz to 10,000 Hz.

We designed a custom electronic circuit to power the microphones and to amplify and filter their output signal (Fig. S2). Each channel uses a dual operational amplifier (OPA2376 Texas Instruments, or LT1884 Linear Technologies) to create a single-supply 3-pole high-pass, 2-pole low-pass filter. DC is first blocked with a high-pass RC filter. The first opamp is non-inverting and applies a 630x gain with an additional capacitor in series at the bottom of the resistive divider to provide a second high-pass pole. Both low-pass poles are implemented in the second opamp, which is configured as a voltage-controlled voltage-source (VCVS) Butterworth filter with 1.6x of gain. A capacitor is added to the bottom of the second opamp's resistive divider to provide the third high-pass pole and block any DC offset. The gain is then set by the ratios between the R4 and R5 resistors, and can be easily changed to

accommodate the intensity of sounds produced by different flies or insects. The low- and high-pass filter cutoff frequencies are determined by the products of five resistor-capacitor pairs.

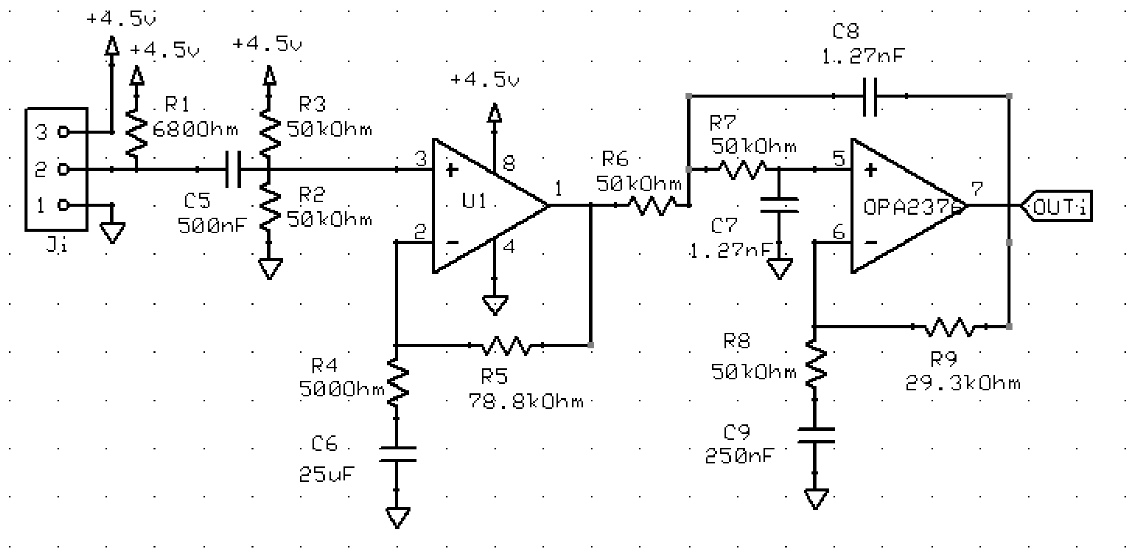


Fig. S2. Amplifier design.

To record *D. melanogaster* song, we set the gain at 1000X and the low and high frequency filters at ~25 and ~2500 Hz. One advantage of our design is that altered cutoff filters or amplification can be accomplished easily by manually changing a few components of the amplifier. Pressure gradient microphones, such as those we used, display sensitivity proportional to frequency, a characteristic that Bennet-Clark purposely corrected for in his amplifier design[3]. Doing so, however, can attenuate high-frequency signals to such an extent that they disappear into the noise. Since we wanted to maintain the flexibility of our amplifier for recording both low and high frequency insect communication, we designed a flat bandpass filter. If needed for accurate amplitude measurements, the frequency response of the microphones can be compensated for later in software.

Low-noise in the circuit is achieved by powering the microphones with a battery, whose level can be monitored with an LED indicator (MAX973, Maxim), and by using one layer of the PC board as a ground plane. Subsequent circuitry runs off of a 5V power supply, which we also regulate for low-noise (LT1129, Linear Tech.). We have found that the noise floor of our electronics is far below the noise floor of the microphone. We have also designed an onboard digital thermometer and hygrometer with a 30-sec update interval (SHT75, Sensirion). Up to five thermometer/hygrometers can be attached to a single board, including four remote devices that allow accurate measurement of temperature and humidity close to the courting flies. Finally, we used a commercial analog-to-digital converter to record the data (NI-USB-6259, National Instruments). The footprint of each channel is 1

square inch on a 2-layer PC board, and the system could be miniaturized by using smaller components, by printing a 4-layer board, and/or by switching to a multiplexed design using the RHA2000 (Intantech.com). In addition, with the current design, a 2-channel version (Supplementary Material) can be powered completely from a 9V battery for use in the field by skipping the external digitizer and by plugging the output directly into the audio input line of a laptop computer.

Courtship chamber

We developed an acrylic platform for 16 chambers (Figure S3). To minimize extraneous vibrations, the courtship chambers are held snugly in the platform by the thumb screws that project through the bottom of each chamber assembly. Two platforms are placed flanking the DAQ, providing 32 recording positions. The entire apparatus, including the DAQ, or just the platforms containing the microphones can be enclosed in an acrylic box, and then mounted on an anti-vibration table.

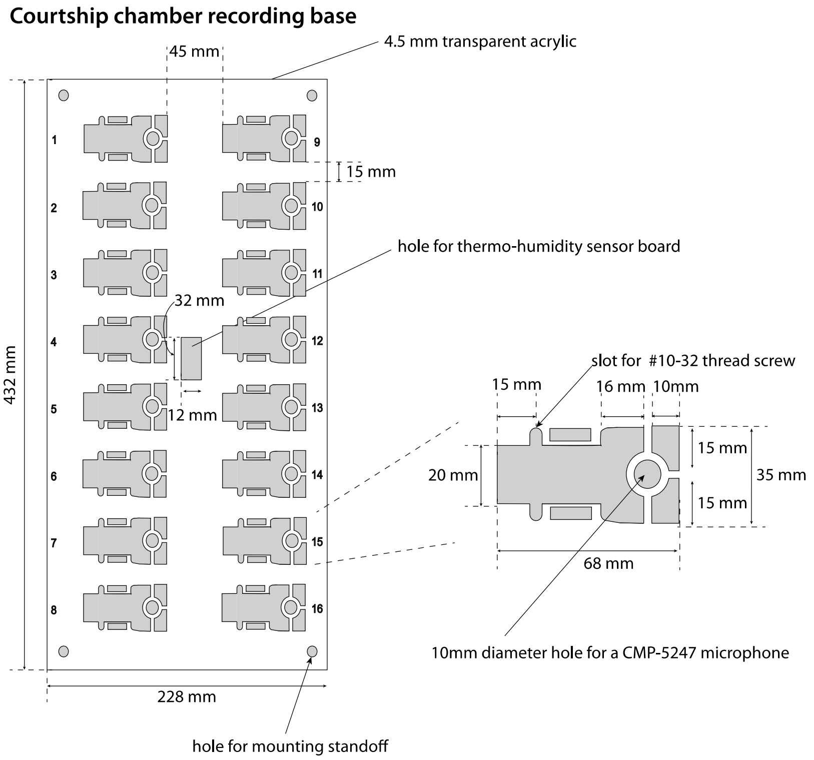


Fig. S3. Acrylic platform.

We discovered that the use of pressure gradient microphones required special adaptations to the recording chambers to minimize the background noise. In particular, chambers were constructed with large air gaps immediately flanking the microphone position, which permits equilibration of air pressure between the front and back of the microphone (Fig. S4). We cut all acrylic for these chambers using a PLS4.75 Universal Laser System. Commercial laser cutters can be purchased

relatively inexpensively, and are often available in campus engineering or similar shops. Alternatively, all of the parts can be milled in acrylic.

Each courtship chamber is assembled from two pieces of cut or milled transparent acrylic, a steel divider, and four thumb screws (Fig. S4). The round arena is 3mm high with a wall sloped at an angle of 45 degrees [4]. The diameter is 5mm at the bottom and 11mm at the top. Nylon mesh (350um opening) is glued to the bottom of the chamber to retain the flies in the arena and to correctly position the steel divider. The top acrylic cover contains two small, staggered holes for loading sequentially a male and a female into the arena on alternative sides of the steel divider. CorelDraw files containing plans for using a laser cutter to cut many chambers from a single piece of acrylic on a laser cutter are provided in file "*designs of chamber_mesh_table .cdr*".

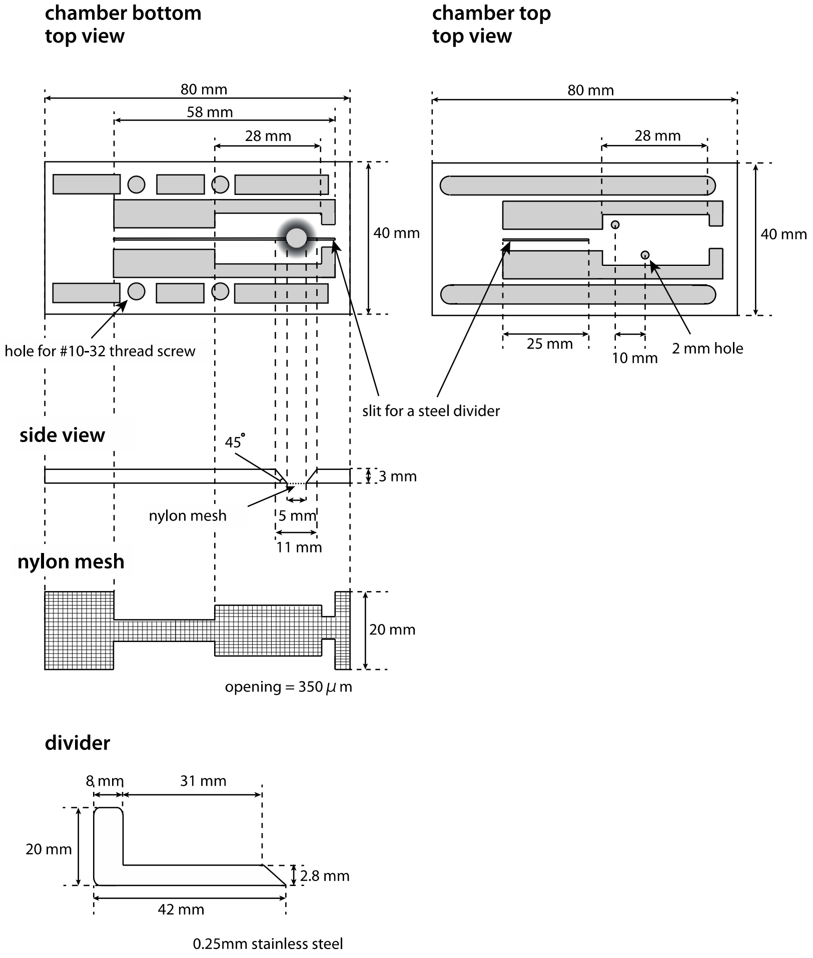


Fig. S4. Courtship chamber parts

<u>Sound shielding</u>

The table is mounted to a breadboard with vibration absorbing feet. The whole apparatus is covered with 12mm thick acrylic box and tightly sealed the gap

with a urethane foam tape to prevent noise. The CorelDraw plans for cutting the acrylic shielding are provided as file "*sound shielding box.cdr"*. As an alternative to the breadboard, the apparatus can be mounted on an air table, such as the TMC 66 Series Table Top CSP. In practice, we set up the entire rig in a dedicated room separated from the rest of the lab by a heavy door.

Pulse and sine song detection with *FlySongSegmenter*

We chose wavelets for pulse detection because wavelets provide excellent temporal resolution for detecting short, discontinuous events, such as pulses. Wavelets of different frequencies can be convolved with a signal to determine how regions of a signal compare with the wavelet. We implemented a small number of simple heuristics to eliminate most false negative and false positive events. Segmentation prior to and after each step of heuristic winnowing is saved by *FlySongSegmenter* (*FSS*) and the results at each stage can be examined to assess the performance of the heuristic winnowing steps. We tuned the parameters of *FSS* for *D. melanogaster* song. For example, we chose the derivative of Gaussian (DoG) wavelet family for *D. melanogaster* pulse detection. The second derivative of this family is called the "Mexican hat" wavelet, which resembles a symmetrical version of a *D. melanogaster* pulse (Fig. 2). However, we found that substituting the DoG wavelets with Daubechies or other "pulse-shaped" wavelets did not yield significantly different results.

For sine song detection, we found that multitaper spectral analysis provided a sensitive measure of periodic signals (such as sine song) even with low signal to noise. The human ear and multitaper analysis work in the frequency domain and, when the cyclical signal of sine song is averaged over multiple cycles, the fourier transform of the recording effectively reduces the noise relative to the signal, allowing detection of faint signals. The excellent sensitivity of multitaper spectral analysis to detect sine song allowed us to detect even sine song of such a low amplitude that it cannot be seen by visual inspection of recordings, although it can be heard by the human ear. The increased sensitivity of multitaper analysis comes at the cost of lower temporal resolution than wavelet analysis, and we indeed observed slightly lower accuracy in estimating sine train length relative to human annotation (Fig. 3e). To improve sine detection at the boundaries between sine and pulse song, we masked the pulse events (we set the values in these regions to 0) and performed multitaper spectral analysis on this masked song. *FSS* masks using *Pulses.ModelCull2* by default, but any of the stages of pulse estimation can be user-defined for masking.

Details of pulse detection algorithm in *FlySongSegmenter*

The parameters used in *FSS* have been optimized for detection of sine and pulse song for *D. melanogaster* song recorded in our laboratories. It is likely to work "out of the box" for other labs. Nonetheless, multiple options can be defined in the file params.m to optimize song segmentation. In addition, these options can be

modified to segment song from some other *Drosophila* species. Here we discuss what we have found to be the most important parameters for pulse segmentation.

1) Parameter *fc* (wavelet scales) defines the scales (and is input as frequencies in Hz) over which *FSS* performs the wavelet transformation. The wavelet transformation finds the best match at each point of the signal to a particular wavelet within the DoG family. The output of this is a single vector (the same length as the input signal itself) containing the maximum coefficients for any wavelet family member or scale – this vector is called *cmhSong*. We recommend searching over the frequency range 100-700Hz, in steps of 25Hz. The program saves the frequency (of those used) that best matches the center of each pulse in an array called *Pulses.Wavelet.fcmx*.
2) Parameter *pWid* is the approximate width of a *D. melanogaster* pulse in sample points. Since we have found that pulse shapes are reasonably stereotyped (Fig 4a), this is a simple value to specify (default = 4ms). *pWid* is used to define a window within which to test for local maxima.
3) Parameter *minIPI* defines the minimum IPI expected in the data (default = 10ms). *minIPI* defines a window within which the code searches for pulse peaks by identifying a peak in the smoothed maximum wavelet coefficients isolated by a trough in the smoothed maximum wavelet coefficients on either side. When the peak to trough distance is greater than a user-defined parameter called *thresh* (defined below), the pulse is accepted. In general, *minIPI*/2 should roughly equal *pWid*.
4) Parameter *thresh* defines the minimum peak to trough distance for the smoothed maximum wavelet coefficients.

Pulses that pass the initial identification based on the wavelet fit (*Pulses.Wavelet*) are passed through three rounds of winnowing. First, pulses with amplitude lower than the user defined parameter *minAmplitude* are removed, resulting in an array of putative pulses called *Pulses.AmpCull*. Second, pulses are winnowed based on parameter *maxIPI* to eliminate singlet events, which results in an array called *Pulses.IPICull*. All three structures are saved and returned to the user. Further model-based winnowing, described in the next section, can be performed in an identical manner on each (or all) of these three arrays.

Estimation and use of pulse models

Detection of pulse song using continuous wavelet transform failed to detect very few pulses, resulting in few false negative calls (Fig. 3). However, the results of the raw continuous wavelet transform and even several heuristic winnowing steps resulted often in many false positive events detected. Most of these false positive events probably reflect other sounds made by flies in the chamber, including walking, grooming, and jumping, and these other events tend to have different spectral characteristics. We reasoned that a model-based approach to winnowing pulses that were identified by the continuous wavelet transform might provide some discrimination between true pulses and non-pulse events. We therefore

constructed models of pulse song from a large sample of putative pulses reported by *Pulses.IPICull*. We then estimated the log likelihood ratio that each pulse in a test song fit the pulse model compared with a simple model of noise. This modeling required consideration of several aspects of the biophysics of the singing fly.

The singing fly wing is a dipole source with some unusual acoustic properties [5, 6]. First, the amplitude of courtship song recorded by pressure gradient microphones is influenced both by the distance of the wing from the microphone and the orientation of the fly relative to the microphone [5, 6]. Second, the phase of individual song events depends on whether the (directional) microphone is positioned in front of or behind the fly. These phase differences were corrected by inverting pulses with negative maximum amplitude. Third, in flying flies, the second harmonic can exhibit more power than the fundamental frequency along the long axis of the wing [6]. We are not aware of any published reports on sound radiation around a singing fly, but we have found evidence for song pulses that look more like pulses expected if the second harmonic dominated occasionally. While we have not confirmed that these occur when the microphone is positioned at approximately 90° to the long axis of the body, we did treat these events separately during construction of pulse models.

Pulse models were estimated first by aligning all individual pulse events and rescaling by their root mean square (RMS). Pulses with negative maximum absolute amplitudes were inverted. Approximately 17% of pulses fit the initial model poorly. Upon closer examination, it appeared that the carrier frequency of these outlier pulses was approximately double the carrier frequency of the initial model. Therefore, pulse events were pooled as either first or second-harmonic events and separate models were built for each harmonic (Fig. S5).

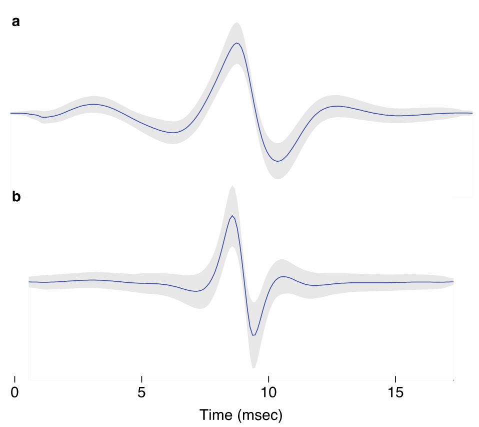


Figure S5. Pulse models estimated from 262,466 individual pulse events. (a) The first harmonic model was generated from the 225,168 events that best fit a single-harmonic model. (b) The second harmonic model was generated from 37,298 events that best fit the second-harmonic model.

A noise model was calculated as an event with mean 0 and the same standard deviation as the pulse model. We then calculated the log likelihood ratio (log(Lik(event|pulse_model) – log(Lik(event/noise_model))) for each event identified in *Pulses.AmpCull* and in *Pulses.IPICull*, resulting in *Pulses.ModelCull* and *Pulses.ModelCull2*, respectively. *Pulses.ModelCull* events with a log likelihood ratio greater than 0 were taken as "true pulses" and included in all subsequent analyses of pulses.

<u>Correlations between song parameters</u>

Pulse train lengths were calculated by collecting consecutive pulses with IPIs within the 99% confidence intervals for IPIs defined by the Gaussian mixture models discussed in the main text (Fig. 4b). Sine trains were estimated directly from the consecutive temporal windows of sine song detected by *FlySongSegmenter.* We tested for correlations between all possible comparisons of pulse and sine carrier frequency, inter-pulse interval, and sine and pulse train lengths (Table S1).

Table S1. Spearman's rank correlation coefficient (*rho* in upper diagonal) and uncorrected P-values (in lower diagonal).

| | Pulse Carrier Frequence | Sine Carrier Frequency | Inter-pulse Interval | Sine Train Length | Pulse Train Length |
|---|---|---|---|---|---|
| Pulse Carrier Frequence | | -0.1568 | -0.1581 | -0.4603 | -0.2833 |
| Sine Carrier Frequency | 0.1853 | | -0.2598 | 0.3993 | 0.3928 |
| Inter-pulse Interval | 0.1977 | 0.0324 | | 0.0205 | -0.2206 |
| Sine Train Length | 0.0000 | 0.0004 | 0.8680 | | 0.4667 |
| Pulse Train Length | 0.0159 | 0.0006 | 0.0729 | 0.0000 | |

<u>Temporal dynamics of song statistics</u>

We assessed periodicity in song statistics with the Lomb-Scargle Periodogram [7, 8] using the Matlab code written by Christos Saragiotis (www.mathworks.com/matlabcentral/fileexchange/22215-lomb-normalized-periodogram). This approach provides accurate estimates of periodicity in time series data in which samples are spaced unequally.

To assess the power of the Lomb-Scargle periodogram coupled with sample sizes equivalent to our samples to detect periodicity in the expected range of 0.016 – 0.022 Hz [9, 10] if it existed, we performed simulations. We resampled the time stamps of the IPIs in our songs and simulated periodic variation in the IPI of 0.018 Hz, which is equivalent approximately to a period of 55 sec. To assess the importance of the signal to noise ratio (SNR) to our ability to detect this periodicity,

we simulated that the IPIs were sampled from a period distribution with added Gaussian noise in the SNR range of 0.1 to 2, in steps of 0.1. Summed over all simulated recordings, the Lomb-Scargle periodogram found significant power in the range of 0.016 – 0.022 Hz in more than 80% of recordings with a SNR > 1 (Fig. S6). We conclude that we could have detected periodicity in the IPI on the order of second or minutes in our data if the SNR of the periodicity was at least 1.

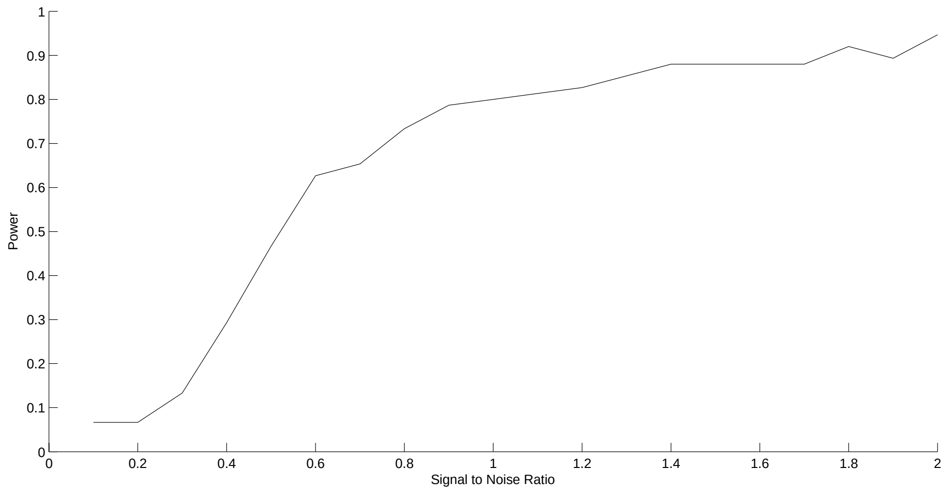


Figure S6. Power to detect significant peaks (P < 0.05) in the range of 0.016 – 0.022 Hz in the Lomb-Scargle periodograms from simulated data as the SNR varies from 0.1 to 2.

<u>Periodicity in sine song fundamental frequency</u>

We also tested for periodicity in sine song fundamental frequency using the Lomb-Scargle periodogram. Many songs displayed unique periodicities (e.g. Fig. S7), but we did not detect any obvious periodicities that were shared across individuals. Sine song fundamental frequency does show interesting patterned modulation (Fig. 6), and we suspect that this modulation leads to significant unique signals of periodicity in many of the songs.

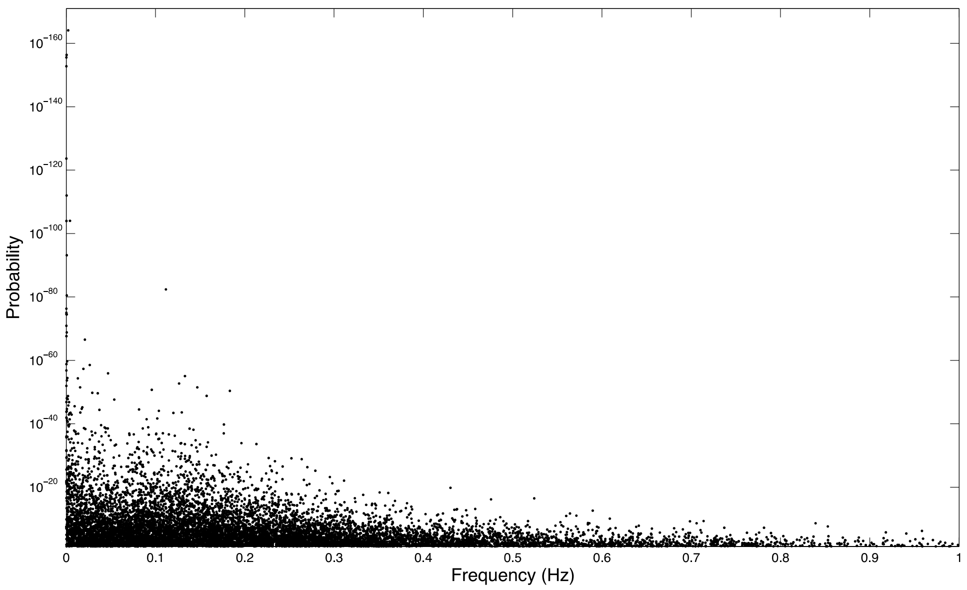


Figure S7. P-values of local peaks in Lomb-Scargle periodograms of sine song fundamental frequency for 75 recordings of *D. melanogaster* over the range of 0 to 1 Hz.

To determine our power to detect song-wide periodic rhythms in sine song, we performed simulations identical to the simulations for interpulse interval described above, except that the event times were taken from the actual sine song events detected in our dataset and we simulated a periodic rhythm of 0.2 Hz, corresponding to a period of 5 seconds. An example of a simulated data set with a SNR of 0.5 is shown in Fig. S8. The significance of peaks from the Lomb-Scargle periodograms of simulated datasets are shown in Fig. S9. For the sine song simulations, Lomb-Scargle periodograms identified significant ($P < 0.05$) peaks between 0.18 to 0.22 HZ in all simulations, from SNR = 0.1 to SNR = 2. Thus, our sine song datasets were sufficiently large that we could have detected significant periodicity with the Lomb-Scargle periodogram even if the SNR of the periodicity was as low as 0.1.

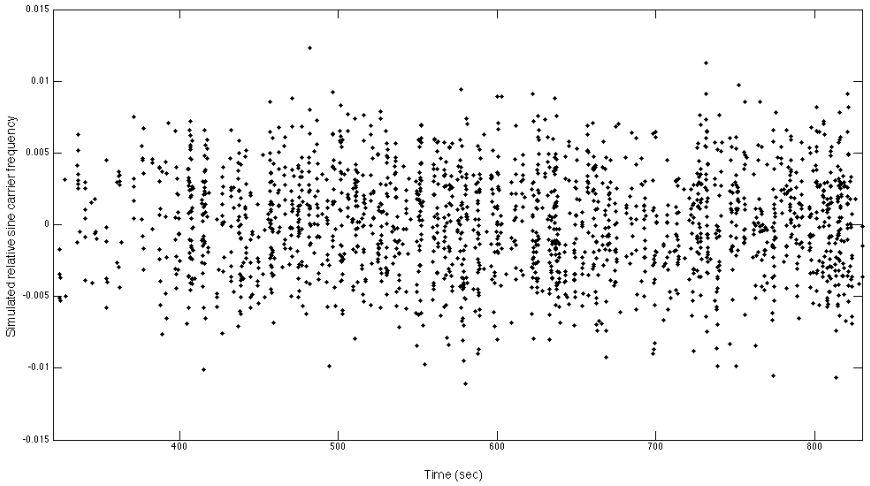


Fig. S8. Simulated data set of a periodic cycle of 0.2Hz with a SNR = 1.

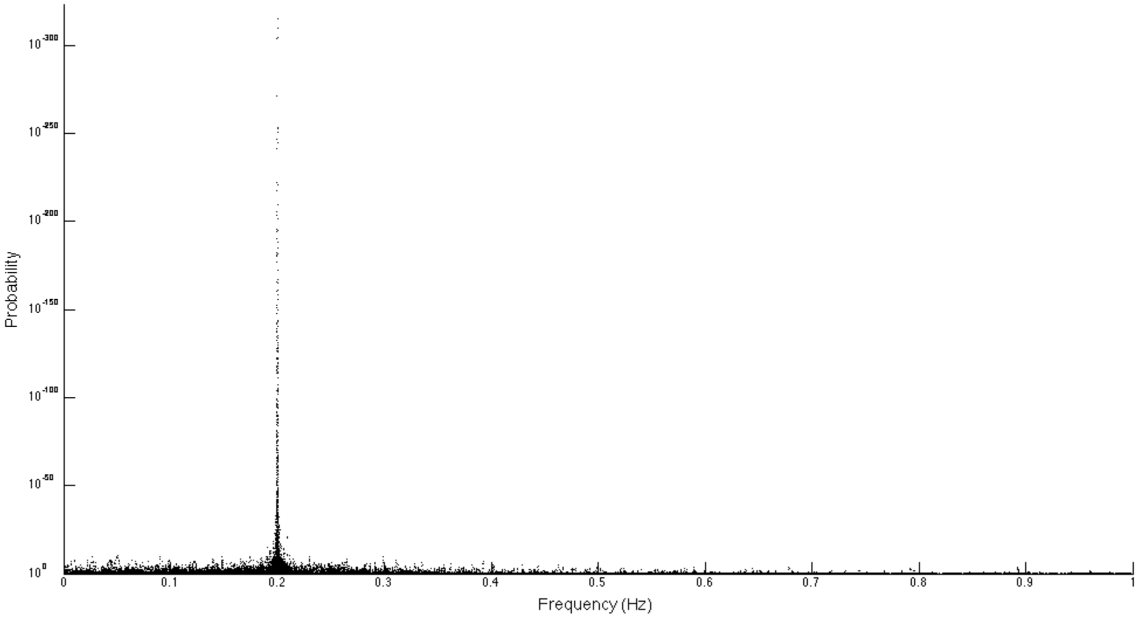


Fig. S9. P-values for local peaks in the Lomb-Scargle periodograms over all simulated song in the range of 0 – 1 Hz, with a simulated periodicity of 0.2 Hz and a SNR = 1.

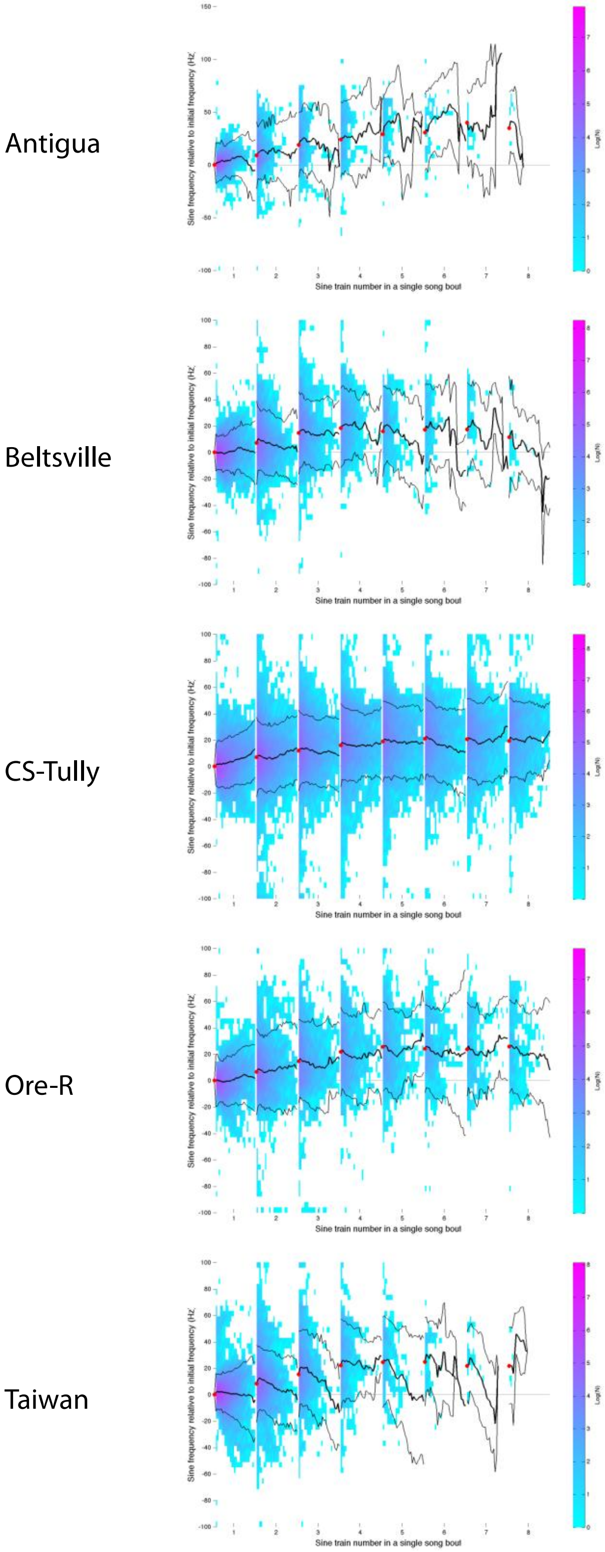


Fig. S10. Patterns of sine song carrier frequency across trains within a song bout across five strains of *D. melanogaster.*

**References**


*1. Bennet-Clark HC: A particle velocity microphone for the song of small insects and other acoustic measurements. J exp Biol 1984, 108:459-463.*

*2. Gopfert MC, Robert D: Active auditory mechanics in mosquitoes. Proc Biol Sci 2001, 268(1465):333-339.*

*3. Bennet-Clark HC: Microphone and pre-amplifier for recording courtship song of Drosophila. Drosophila Information Service 1972, 49:127-128.*

*4. Simon JC, Dickinson MH: A new chamber for studying the behavior of Drosophila. PLoS ONE 2010, 5(1):e8793.*

*5. Bennet-Clark HC: Acoustics of insect song. Nature 1971, 234:255-259.*

*6. Sueur J, Tuck EJ, Robert D: Sound radiation around a flying fly. J Acoust Soc Am 2005, 118(1):530-538.*

*7. Lomb NR: Least-squares frequency analysis of unequally spaced data. Astrophysics and Space Science 1976, 39:447-462.*

*8. Scargle JD: Studies in astronomical time series analysis. II - Statistical aspects of spectral analysis of unevenly spaced data. Astrophysical Journal, Part 1 1982, 263:835-853.*

*9. Kyriacou CP, Hall JC: Circadian rhythm mutation in Drosophila melanogaster affect short-term fluctuation in the male's courtship song. PNAS 1980, 77:6729-6733.*

*10. Kyriacou CP, Hall JC: Spectral analysis of Drosophila courtship song rhythms. Anim Behav 1989, 37:850-859.*
